# Insights into the activity of nickel boride/nickel heterostructures for efficient methanol electrooxidation

**DOI:** 10.1038/s41467-022-32443-5

**Published:** 2022-08-06

**Authors:** Yanbin Qi, Yue Zhang, Li Yang, Yuhan Zhao, Yihua Zhu, Hongliang Jiang, Chunzhong Li

**Affiliations:** 1 Key Laboratory for Ultrafine Materials of Ministry of Education, School of Chemical Engineering, East China University of Science and Technology, Shanghai, 200237 China; 2 Shanghai Engineering Research Center of Hierarchical Nanomaterials, School of Materials Science and Engineering, East China University of Science and Technology, Shanghai, 200237 China; 3 Institutes of Physical Science and Information Technology, Anhui University, Hefei, 230601 China

**Keywords:** Energy, Chemical hydrogen storage, Electrocatalysis

## Abstract

Designing efficient catalysts and understanding the underlying mechanisms for anodic nucleophile electrooxidation are central to the advancement of electrochemically-driven technologies. Here, a heterostructure of nickel boride/nickel catalyst is developed to enable methanol electrooxidation into formate with a Faradaic efficiency of nearly 100%. Operando electrochemical impedance spectroscopy and in situ Raman spectroscopy are applied to understand the influence of methanol concentration in the methanol oxidation reaction. High concentrations of methanol inhibit the phase transition of the electrocatalyst to high-valent electro-oxidation products, and electrophilic oxygen species (O* or OH*) formed on the electrocatalyst are considered to be the catalytically active species. Additional mechanistic investigation with density functional theory calculations reveals that the potential-determining step, the formation of *CH_2_O, occurs most favorably on the nickel boride/nickel heterostructure rather than on nickel boride and nickel. These results are highly instructive for the study of other nucleophile-based approaches to electrooxidation reactions and organic electrosynthesis.

## Abstract (web summary)

Understanding the role of active sites in electrooxidation reactions is important yet challenging. Here, the authors use operando spectroscopies to monitor the effect of methanol concentration on Ni_3_B/Ni heterostructures during formate production.

## Introduction

Electrosynthesis powered by renewable electricity has emerged as a potentially sustainable and environmentally benign route for the production of chemicals and fuels^1,2^. In most cases, cathodic electrochemical reduction reactions, typically including hydrogen evolution^3–5^, O_2_^6,7^ and CO_2_ electroreduction^8,9^ as well as organic electrocatalytic hydrogenation^10^, are paired with anodic oxygen evolution reaction (OER). The O_2_ product of OER holds little economic value^11^. Besides, thermodynamic analysis suggests that approximately 90% of the electricity driving these above electrosynthesis processes are consumed by the OER^12^. In other words, anodic OER is main efficiency bottleneck for these processes. In addition to accelerate the OER, electrooxidation reactions of organics such as methanol^13^, urea^14^, 5-hydroxymethylfurfural^15,16^, furfural^17^ and glucose^18^ have been proposed to replace sluggish OER. These organic electrooxidation reactions possess lower onset potentials than OER, thereby reducing the cost of electrolysis. Meanwhile, value-added products can also be obtained at the anode^17^; or the purpose of degrading wastewater can be achieved^19^.

These organic electrooxidation reactions are also called as nucleophile oxidation reactions (NOR)^20^. During the reactions, nucleophilic groups containing active hydrogen atoms in these organic substrates undergo dehydrogenation processes to form oxygen-rich products. Nickel-based electrocatalysts have been demonstrated as potential candidates for NOR^21,22^. However, much ambiguity is present regarding the mechanism of NOR. A typical work reported by Wang’s group proposed that NOR is a two-step, one-electron reaction including an electrogenerated catalyst dehydrogenation reaction from lattice hydroxyl to electron-deficient lattice oxygen and a spontaneous nucleophile dehydrogenation reaction on model Ni(OH)_2_ catalysts^20^. Compared to Ni(OH)_2_, some recent studies have shown that nickel non-hydroxides exhibit better performance^23–25^. Nevertheless, the surface species transformation of metallic catalysts often occurs during electrooxidation, leading to much controversy regarding the activity origin^20^. Some researches suggested that the oxyhydroxides in situ generated from the non-(hydro)oxide pre-catalysts are identified as a catalytically active phase^26,27^. Another statement is that the NOR proceeds via adsorbate oxidation mechanism pathway^28^. Designing efficient electrocatalyst and clarifying its NOR mechanism are essential for the development of nucleophile electrooxidation.

In this work, we focused on a typical nucleophile, methanol, in consideration of the advantages of its simple structure and high stability under alkaline condition. A heterostructure of nickel boride/nickel catalyst was successfully developed for methanol electrooxidation reaction (MOR), delivering nearly 100% formate Faradaic efficiency and long-term stability. Operando electrochemical impedance spectroscopy (EIS) was carried out to track the MOR and OER processes. It was revealed that the OER occurred on the oxide layer formed after catalyst electrooxidation, while the MOR process was not accompanied by observable catalyst electrooxidation when methanol concentration is high, which were also evidenced by in situ Raman spectroscopy. The electrooxidation of catalyst could completely be suppressed in high concentrations of methanol. The electrophilic OH* or O* was produced as the potential increases, and immediately the methanol molecule underwent a spontaneous nucleophile dehydrogenation. At low methanol concentrations, the high-valent products formed by catalyst electrooxidation are catalytically active species for MOR. The proposed mechanism well explained the effect of the presence of methanol on the selectivity towards the anodic reactions, catalyst passivation and catalyst electrooxidation. Furthermore, in situ attenuated total reflection surface enhanced infrared absorption spectroscopy (ATR-SEIRAS) measurement was performed to probe reaction intermediates and identify the methanol electrooxidation pathway. Density functional theory (DFT) calculations were also employed to understand the reasons for efficient methanol electrooxidation on the nickel boride/nickel heterostructure.

## Results

### Catalyst preparation and characterization

**Fig. 1:**
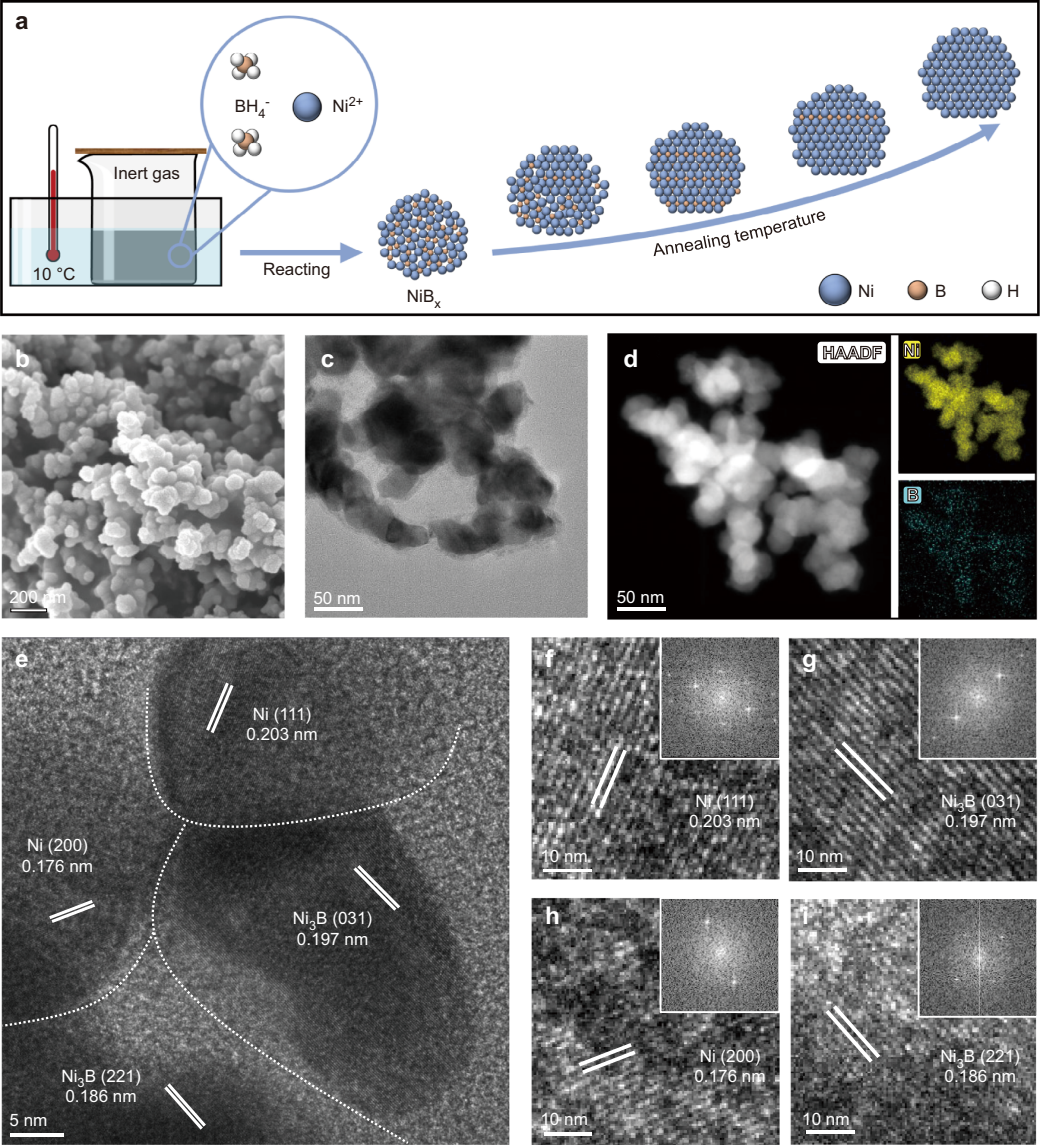
Preparation and Characterizations of the nickel boride/nickel catalysts. **a** Schematic diagram of the catalyst preparation. **b** SEM image of the prepared NiB-400 catalyst. **c** TEM image of the prepared NiB-400 catalyst. **d** STEM-EDX images of NiB-400 catalyst. **e** HRTEM image of the prepared NiB-400 catalyst. **f**–**i** Local enlargement of the four regions in **e**.

The nickel boride/nickel catalyst was prepared by reacting NaBH_4_ with nickel nitrate, followed by annealing in H_2_-Ar atmosphere (Fig. 1a, see details in methods)^29^. The NaBH_4_ acted as a reducing agent to reduce the nickel ion to form nickel boride (NiB_x_). The nickel nitrate solution was changed from green to black after the addition of NaBH_4_, indicating that Ni^2+^ was reduced. NiB_x_ was used as a precursor, which was further transformed into nickel boride/nickel (NiB_x_/Ni) after annealing at different temperature in H_2_-Ar atmosphere. The discussion hereafter mainly focused on the sample annealed at 400 °C (denoted as NiB-400).

X-ray powder diffraction (XRD) was conducted to investigate the phase composition of the samples (Supplementary Fig. 1–4). The XRD pattern of NiB-400 is shown in Supplementary Fig. 1. The main peaks are assigned to orthorhombic Ni_3_B and face-centered cubic Ni. Weak peaks assigned to cubic B_2_O_3_ could also be observed. Unannealed precursor (NiB_x_) is amorphous, and the crystalline phases appear when annealing temperature is above 300 °C (Supplementary Fig. 2). Inductive Coupled Plasma Emission Spectrometer was utilized to determine the elemental content of NiB_x_, and the result shows that the content ratio of Ni to B is about 2.56:1 (Supplementary Table 1). The surface chemical state was investigated by X-ray photoelectron spectroscopy (XPS). As shown in Ni 2*p* spectrum of NiB-400 (Supplementary Fig. 5a), the peak at 852.8 eV is attributed to Ni^0^ in metal nickel and Ni_3_B. The peak at 856.9 eV is assigned to oxidized Ni. The peaks at 188.2 eV and 192.9 eV in the B 1 *s* spectrum were attributed to boride and oxidized boron, respectively (Supplementary Fig. 5b). XPS spectra of as-prepared Ni_3_B and Ni are shown in Supplementary Fig. 6, 7. Scanning electron microscopy (SEM) image (Fig. 1b) and transmission electron microscopy (TEM) images (Fig. 1c, Supplementary Fig. 8, 9) show that the as-prepared nickel boride/nickel, nickel boride and nickel exhibit nanoparticle morphology. Scanning TEM-energy dispersive X-ray (STEM-EDX) shows the homogeneous distribution of Ni element and B element (Fig. 1d). STEM-EDX and high-resolution transmission electron microscopy (HRTEM) reveal the existence of numerous interfaces in NiB-400 (Fig. 1d, e), which is consistent with previous report^29^. Figure 1f–i are enlarged views of the four regions in Fig. 1e. The interplanar spacings of the four regions are measured to be 0.203, 0.197, 0.176 and 0.186 nm, corresponding to the (111) crystal plane of fcc Ni, the (031) crystal plane of orthorhombic Ni_3_B, the (200) crystal plane of fcc Ni and the (221) plane of orthorhombic Ni_3_B (Supplementary Fig. 10).

### Performance of nickel boride/nickel catalyst for methanol oxidation

**Fig. 2:**
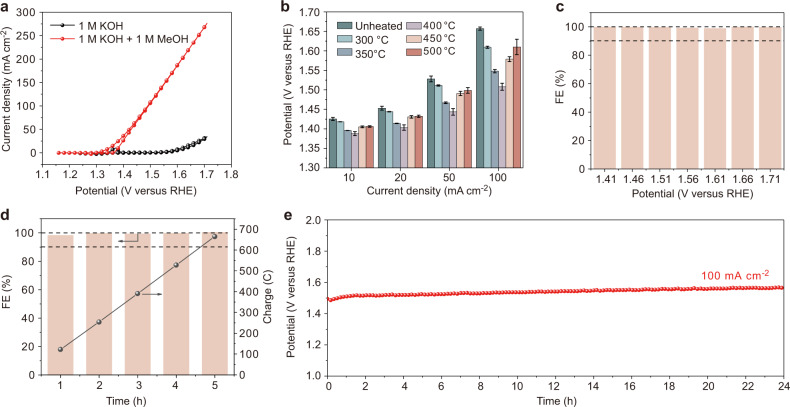
Electrocatalytic performance of nickel boride/nickel catalyst for MOR. **a** CV curves of NiB-400 in 1.0 M KOH electrolyte with and without 1 M methanol. **b** Comparison of the applied potentials required to achieve varied current densities in 1.0 M KOH with 1 M MeOH electrolyte for samples annealed at different temperatures. **c** Faradaic efficiency of formate obtained after 1 h of electrolysis at different potentials in 1.0 M KOH electrolyte with 1 M MeOH. **d** Faradaic efficiency of formate obtained after 5 h continuous electrolysis at 1.61 V in 1.0 M KOH electrolyte with 1 M MeOH. **e** CP profile at a current density of 100 mA cm^−2^. Source data are provided as a Source Data file.

Figure 2a shows the cyclic voltammetry (CV) curves obtained in 1 M KOH electrolyte and 1 M KOH + 1 M methanol electrolyte. When methanol is added to the KOH electrolyte, the onset potential of the oxidation reaction is significantly reduced, and the potential required for a current density of 10 mA cm^−2^ is reduced by about 235 mV. By comparing the potentials required to achieve the same current densities for samples annealed at different temperatures, it is found that the sample annealed at 400 °C exhibits the best performance (Fig. 2b). Compared with the recently reported Ni-based electrocatalysts, NiB-400 exhibited superior performance for MOR (Supplementary Table 2). The electrochemical double-layer capacitance (C_dl_) of samples annealed at different temperatures shows a trend of decreasing with increasing annealing temperature, indicating the high intrinsic activity of NiB-400 (Supplementary Fig. 11). Combined with the XRD patterns (Supplementary Fig. 2), it can be seen that the improvement of performance is synchronized with the generation of Ni and Ni_3_B crystalline phases after annealing. With the further increase of the annealing temperature, the content of metal Ni increases due to the transformation of Ni_3_B into Ni^29^, and the performance decreases accordingly. Considering that the XRD pattern showing that the material contains a small amount of B_2_O_3_ (Supplementary Fig. 1), the methanol oxidation performance of pure B_2_O_3_ and clean glassy carbon electrode were tested. The pure B_2_O_3_ exhibits the same cubic phase as the B_2_O_3_ in NiB-400 (Supplementary Fig. 12). The performance of pure B_2_O_3_ and clean glassy carbon on methanol electrooxidation is extremely poor (Supplementary Fig. 13). Further, the performance of Ni_3_B and metal Ni on methanol oxidation was tested. The performance of Ni_3_B and metal Ni is far inferior to that of NiB-400 (Fig. 2b, Supplementary Fig. 14), which indicates that the catalytic activity may originate from the nickel boride/nickel heterostructure rather than pure Ni_3_B or Ni. Faradaic efficiency (FE) of formate obtained after 1 h of chronoamperometry (CA) test at 1.41–1.71 V in 1 M KOH + 1 M methanol electrolyte is very close to 100%, indicating high selectivity to formate product (Fig. 2c, Supplementary Fig. 15). During the continuous reaction at 1.61 V for 5 h, the Faradaic efficiency of formate is maintained around 100% (Fig. 2d). Chronopotentiometry (CP) measurement shows that an additional potential of only 0.07 V, above the initial 1.49 V, is required to maintain a current density of 100 mA cm^−2^ for 24 h (Fig. 2e). After compensating for the voltage drop, a large current density of 500 mA cm^−2^ can be achieved at 1.54 V using NiB-400 as the MOR electrocatalyst (Supplementary Fig. 16), which meets the requirement of high current density for potentially industrial applications. After MOR, NiB-400 maintains its original morphology and crystalline phase (Supplementary Fig. 17). The Ni 2*p* XPS spectra of NiB-400 after OER and MOR are shown in Supplementary Fig. 18.

### Tracking of electrochemical processes and evolution of the catalyst

**Fig. 3:**
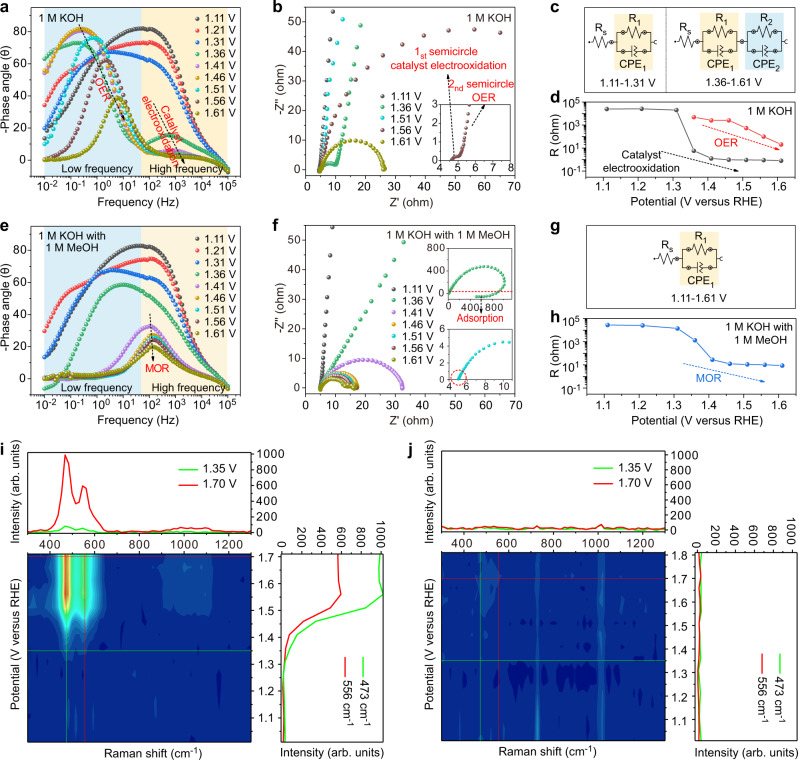
Operando electrochemical impedance spectroscopy characterization. **a** Bode plots and **b** Nyquist plots of NiB-400 for OER in different potentials. **c** Equivalent circuit models for OER. **d** Correlation of the equivalent resistances (R_1_ and R_2_) and potentials for NiB-400 during OER. **e** Bode plots. **f** Nyquist plots of NiB-400 for MOR in different potentials. **g** Equivalent circuit models for MOR. **h** Correlation of the equivalent resistance and potentials for NiB-400 during MOR. In situ Raman spectra of NiB-400 in OER **i** and in MOR **j** processes. Source data are provided as a Source Data file.

Operando electrochemical impedance spectroscopy (EIS) recorded at various potentials was utilized to track the electrochemical processes of MOR and OER. In 1 M KOH electrolyte (Fig. 3a), the response appearing in the high frequency region after 1.36 V is associated with the oxidation of the electrode inner^30^. The response appearing in the low frequency region is related to the nonhomogeneous charge distribution caused by surface oxidized species^30,31^. It can be seen that the low-frequency interface appears with the electrooxidation of the catalyst, indicating that the low-frequency interface is between the diffuse double layer (DDL) and the oxidized layer formed by the electrooxidation of the catalyst. OER occurs on this oxidized layer formed by the electrooxidation of the electrocatalyst^20^. Correspondingly, the Nyquist plots show two semicircles after 1.36 V (Fig. 3b). The semicircles in the high and low frequency regions correspond to the electrooxidation of the catalyst and the OER, respectively. The equivalent circuit models for the operando EIS measured in 1 M KOH are shown in Fig. 2c. Before 1.36 V, the equivalent resistance R_1_ is large, indicating that the charge transfer is extremely weak. After reaching 1.36 V, R_1_ suddenly decreases significantly, indicating the onset of catalyst electrooxidation, while the equivalent resistance R_2_, reflecting the OER, appears and decreases with increasing potential. However, in 1 M KOH electrolyte containing 1 M methanol (Fig. 3e), only one response in the frequency region around 100 Hz is displayed in the Bode plot after 1.36 V. No phase angle peak in low frequency and higher frequency indicates the difference in the reaction interface and no electrooxidation of the catalyst. The MOR in 1 M KOH + 1 M methanol electrolyte does not occur on the surface of oxidized layer (NiOOH, NiO_x_) formed by the electrooxidation of electrocatalyst, but directly occurs between the DDL and the surface of the catalyst. In the Nyquist plot obtained in 1 M KOH containing 1 M methanol electrolyte (Fig. 3f), only a semicircle corresponding to the MOR reaction is shown after 1.36 V. Interestingly, Nyquist plot shows that the real part at low frequency contracted at 1.36 V, which is due to the adsorption of electroactive species. The equivalent circuit and equivalent resistance of the MOR process are shown in Fig. 3g–h. Both in 1 M KOH electrolyte and 1 M KOH + 1 M methanol electrolyte, the equivalent resistance begins to drop sharply at 1.36 V (Fig. 3d, h, Supplementary Table 3-4), suggesting that MOR and OER may involve some of the same intermediate species. However, the same intermediate species of the two reactions subsequently undergo different changes, resulting in differences in the reaction interfaces of MOR and OER.

In situ Raman spectroscopy was used to investigate the changes in catalyst during the OER and MOR processes (Fig. 3i–j). The ex situ Raman spectrum of 1 M KOH + 1 M MeOH electrolyte is shown in Supplementary Fig. 19. In the OER process (1 M KOH electrolyte), two signal peaks appear at ~473 cm^−1^ and ~556 cm^−1^ at 1.35 V, corresponding to the bending vibration and stretching vibration of Ni^3+^-O of γ-NiOOH, respectively (Fig. 3i). At high potentials, the broad peak observed in the 800–1150 cm^−1^ wavenumber region is attributed to the formation of NiOO^−^^32,33^. However, no such features can be found in the MOR process (1 M KOH + 1 M MeOH electrolyte), which means no phase transformation to NiOOH occurs with the existence of high concentrations of methanol (Fig. 3j). The results of in situ Raman spectroscopy are consistent with the results of operando EIS. Therefore, it may be inaccurate to assume that the MOR is an process catalyzed by NiOOH in the presence of high concentrations of methanol.

### Effect of methanol concentration on the reaction process

**Fig. 4:**
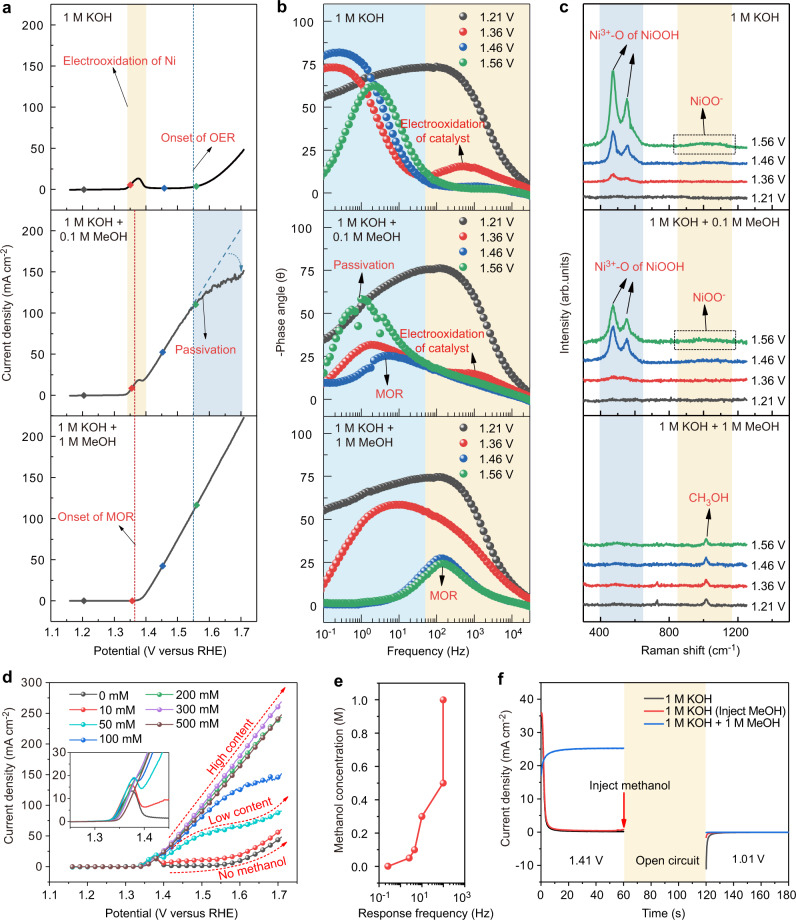
Effect of methanol concentration. **a** LSV curves (Scan rate was set as 20 mV/s), **b** Bode plots of operando EIS and (**c**) in situ Raman spectra measured in 1 M KOH, 1 M KOH + 0.1 M MeOH and 1 M KOH + 1 M MeOH. **d** LSV curves in electrolytes with different concentrations of methanol. (Scan rate was set as 20 mV/s). **e** The response frequency of the reaction at 1.41 V in the Bode plots as a function of methanol concentration. **f** Periodic electrochemical measurement over NiB-400. Source data are provided as a Source Data file.

Methanol concentration has an effect on the shape of the linear sweep voltammetry (LSV) curve. Many Ni-based materials exhibit the similar characteristic^23,34,35^, but the reason for this characteristic has not been further investigated. The change of the shape of LSV curve may reflect the change of reaction process or even reaction mechanism. For this reason, the LSV curves, Bode plots of operando EIS and in situ Raman spectra measured at different methanol concentrations are compared in Fig. 4a–d. The presence of the oxidation peak of Ni^II^ to Ni^III^ in the LSV curve obtained in 1 M KOH + 0.1 M MeOH electrolyte indicates the occurrence of catalyst electrooxidation, which is consistent with that in 1 M KOH (Fig. 4a). Correspondingly, the characteristics of catalyst electrooxidation can be observed in the Bode plots of operando EIS (Fig. 4b) and in situ Raman spectra (Fig. 4c, Supplementary Fig. 20). The current then rises after 1.36 V, indicating the onset of methanol oxidation. However, unlike the case of 1 M MeOH, the response frequency of MOR in the Bode plots is in the low frequency region in the presence of 0.1 M MeOH (Fig. 4b middle), exhibiting similar characteristic to that of OER. This indicates that when the methanol concentration is low, the MOR occurs on the oxide layer formed by the electrooxidation of the catalyst. As shown in Fig. 4e and Supplementary Fig. 21, the response frequency of the reaction in the Bode plots shifts to the higher frequency with the increase of methanol concentration and stabilizes after the methanol concentration reaches 0.5 M, remaining at ~100 Hz. This indicates the change of the catalytic reaction interface from the low frequency interface to the high frequency interface. In addition, the current collapse starts to appear in the LSV curve as the potential increases. As can be seen from Fig. 4b, d, the passivation disappears with further increase of methanol concentration, and it can be found that the passivation phenomenon and the oxidation peak are disappearing simultaneously, which means that the passivation phenomenon is associated with the electrooxidation of the catalyst. The small decrease in current appears in LSV curves after the methanol concentration exceeds 0.3 M stems from the increase in solution resistance (Supplementary Fig. 22). In the Bode plots of EIS, the passivation phenomenon is usually manifested by the abnormal change of the peak position and peak height of the phase angle peak, obvious signal fluctuation or phase angle exceeding 90 degrees, etc. The reason for the passivation phenomenon is that species with poor MOR activity are generated^20^ due to the oxidation of the electrocatalyst and OER begins to compete with MOR^36^ as the potential increases. As shown in Fig. 4f, after applying a potential higher than 1.36 V, methanol was injected into 1 M KOH and kept the open circuit for 60 s. When a potential of 1.01 V is applied, the reduction current is significantly lower (red line) than that without methanol injection (black line). This indicates that methanol can react spontaneously and rapidly with the NiOOH without dependence on potential, leading to the consumption of NiOOH and thus the reduction current is decreased. For the case where methanol is present throughout (blue line), no reduction current is present when a potential of 1.01 V is applied, indicating the inhibitory effect of methanol on NiOOH formation.

All the above-mentioned evidences show that the high concentration of methanol can inhibit the catalyst electrooxidation to NiOOH, making the OER active site lacking, thus reducing the vicious competition between OER and MOR. Therefore, the Faradaic efficiency of formate is increased to nearly 100% with the increase of methanol concentration in the electrolyte (Supplementary Fig. 23).

### Insights into the MOR mechanism

**Fig. 5:**
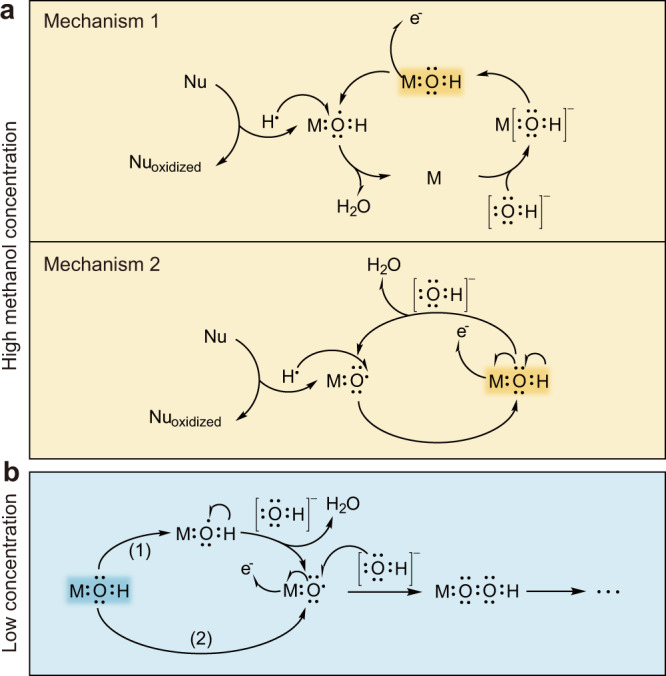
Mechanisms of MOR. **a** Mechanisms of methanol oxidation reaction in high methanol concentration case (Upper: Ni atom loses electrons and the reaction involves electrophilic OH*. Lower: OH* on the catalyst surface loses hydrogen atoms and the reaction involves electrophilic O*). **b** Mechanisms of methanol oxidation reaction in low methanol concentration case ((1) Ni atom loses electrons. (2) OH* loses hydrogen atoms).

Given the discussion above, the statement about MOR is catalyzed by the electrooxidation products of the catalyst such as NiOOH may be inaccurate, since the effect of methanol concentration on the structural changes of the catalyst is ignored. In alkaline electrolyte, the surface of the electrocatalyst adsorbs OH^−^ to form OH*, making the surface appear divalent, which is consistent with the XPS result of the reacted electrocatalyst (Supplementary Fig. 18b). Strong OH* adsorption brings about a phase transition of Ni (oxy)hydroxide, but it becomes different when methanol molecules are present. From the LSV curves, the onset potential of MOR is at about 1.36 V. This potential is consistent with the oxidation potential of Ni^II^ to Ni^III^. For this oxidation process, two possible oxidation pathways are proposed here: (1) Ni atom lost electrons leading to electron deficiency of OH* adjacent to it; (2) Dehydrogenation of OH* adjacent to the Ni atom. These two possible pathways lead to the formation of electrophilic OH* and electrophilic O*, respectively. When the concentration of nucleophilic reagent methanol is high (≥ 0.5 M), the electrophilic oxygen species (OH* or O*) can rapidly capture hydrogen atoms from the methanol molecules, which leads to the oxidation of the methanol molecules while the catalyst eventually returns to its initial state (highlighted by dark colors) and the reaction is not accompanied by electrooxidation products of the catalyst such as NiOOH (Fig. 5a). When the methanol concentration is low (Fig. 5b), the electrophilic oxygen species cannot capture hydrogen atoms from methanol molecules in time, leading to the accumulation of O*. The accumulation of O* induces the occurrence of phase transition to form electrooxidation products of the catalyst such as NiOOH, which is consistent with the results of operando EIS (Fig. 4b) and in situ Raman spectroscopy (Fig. 4c, Supplementary Fig. 20). O* that cannot be consumed in time will combine with OH^−^ in the electrolyte and then form NiOO^−^ species that can be observed in the in situ Raman spectra (Fig. 4c, Supplementary Fig. 20). Thus, for the case of low concentrations of methanol, methanol oxidation can be catalyzed by both electrophilic oxygen species and products of catalyst electrooxidation (i.e., the widely proposed electrochemical-chemical process). Since the electrooxidation layer of the catalyst is also catalytically active for OER, OER competes with MOR when the onset potential of the OER is reached, thus leading to the current collapse (Fig. 4a, d). The presence of high concentrations of methanol is not accompanied by the formation of electrooxidation layer, so the OER is inhibited (Fig. 4a, d).

### DFT calculations

**Fig. 6:**
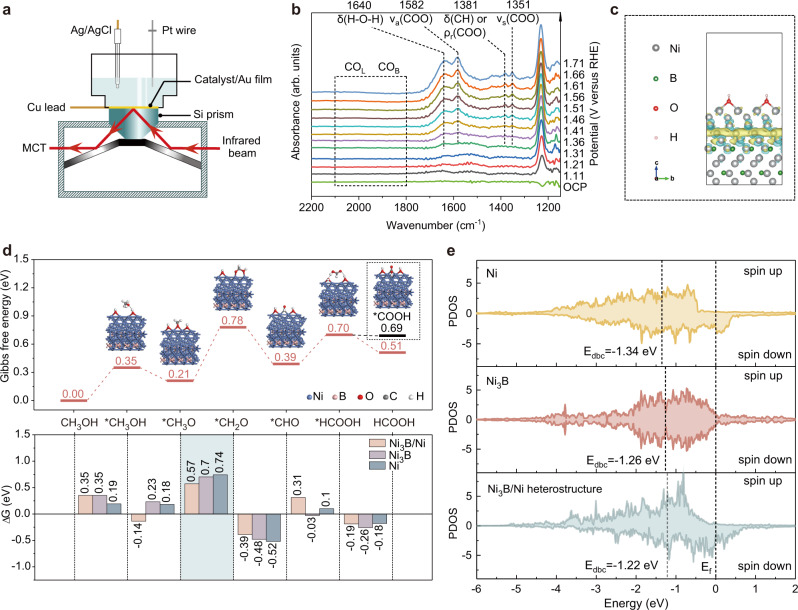
Reaction intermediates detection and DFT calculations. **a** Schematic diagram of the ATR-SEIRAS set-up. **b** ATR-SEIRAS spectra during MOR in 1 M KOH containing 1 M methanol electrolyte. **c** Differential charge density plot of nickel boride/nickel heterostructure. **d** Gibbs free energy diagram of MOR occurring on Ni_3_B(001)/Ni(111) heterostructure (upper) and the change in Gibbs free energy between the steps on different surfaces (lower). **e** Partial density of states (PDOS) of d-projected DOS of Ni active sites for Ni_3_B(001)/Ni(111) heterostructure, Ni_3_B and Ni respectively. Source data are provided as a Source Data file.

In situ ATR-SEIRAS performed in 1 M KOH containing 1 M MeOH was utilized to determine the reaction intermediates to identify the reaction path for assisting subsequent DFT calculations. The schematic diagram of the device used is shown in Fig. 6a. As shown in Fig. 6b, with the onset of the reaction after 1.36 V, some new bands start to appear in the spectra. The band at 1582 cm^−1^ is assigned to *v*_a_(COO), the band at 1381 cm^−1^ to δ(CH) or ρ_r_(COO), and the band at 1352 cm^−1^ to *v*_s_(COO) of formate^37,38^. The band at 1640 cm^−1^ can be assigned to water deformation^37^. No band of bridge-bonded CO(CO_*B*_) or linearly-bonded CO(CO_*L*_) can be observed, indicating that no CO intermediate is involved in the MOR process. Besides, the band of CO_3_^2−^ at around 1400 cm^−1^ cannot be found, indicating that further oxidation after the formation of HCOOH is unfavorable. Therefore, the product of MOR on NiB-400 is formate accompanied by water generation during the process. Based on the results of in situ ATR-SEIRAS, it is proposed that the methanol oxidation pathway on NiB-400 is CH_3_OH → *CH_3_OH → *CH_3_O → *CH_2_O → *CHO → *HCOOH → HCOOH. Unlike the previously reported pathway where the oxidation product is CO_2_^39^, there is no further dehydrogenation of *CHO to generate *CO. According to the observed bands of COO and previous report^13^, it is proposed that the OH^−^ in the electrolyte interacts with *CHO leading to the formation of *HCOOH.

The catalytically active species for MOR were discussed above, but the reason for the higher activity of nickel boride/nickel heterostructure over pure nickel boride and pure nickel is still poor understood. Therefore, DFT calculations were further performed. Based on the discussion of catalytically active species above, the catalyst surfaces with adsorbed OH were constructed. On the basis of the finite strain theory, the most matched Ni_3_B(001)/Ni(111) interface was constructed to demonstrate the heterostructure of nickel boride/nickel^29,40^. In addition, DFT calculations were also performed for other surfaces and DFT-optimized configurations of the favorable adsorbed intermediates for the oxidation of MeOH on different surfaces are depicted in Supplementary Fig. 24. Differential charge density plot (Fig. 6c) and Bader charge analysis (Supplementary Table 5) found that the electrons are transferred from Ni to Ni_3_B in the nickel boride/nickel interface, which could facilitate the subsequent oxidation reaction. The Gibbs free energy profiles for the MOR process on the various surfaces are illustrated in Fig. 6d and supplementary Fig. 25. It can be seen that the potential-determining step for MOR are all contributed by the process of *CH_3_O → *CH_2_O + H^+^ + e^−^ on the four surfaces of Ni_3_B(001)/Ni(111) heterostructure, Ni_3_B(221)/Ni(111) heterostructure, Ni_3_B and Ni. Interestingly, Ni_3_B(001)/Ni(111) heterostructure exhibits the lowest Gibbs energy barrier of 0.57 eV in comparison to 0.74 and 0.70 eV for Ni_3_B and Ni, respectively, indicating that MOR can occur most favorably on the Ni_3_B/Ni surface. The ΔG of the HCOOH generation is −0.19 eV, which is much lower than that for the further oxidization to *COOH intermediate (−0.01 eV), suggesting that the final product of the MOR on Ni_3_B/Ni heterostructure is formic acid rather than carbon dioxide. This is also consistent with the in situ ATR-SEIRAS results. In addition, the produced formic acid can readily exist in the form of formate in alkaline medium, spontaneously making it difficult to be further oxidized. The partial density of states (PDOS) for the adsorption behavior of *CH_2_O intermediate on Ni_3_B(001)/Ni(111) heterostructure, Ni_3_B, and Ni structures were also calculated. As shown in Fig. 6e, the d-band centers of Ni_3_B/Ni heterostructure, Ni_3_B, and Ni are separetly −1.22, −1.26, and −1.34 eV. Obviously, the value of Ni_3_B/Ni heterostructure is most closely approaching to the Fermi energy level, which endows the strongest adsorption capacity for *CH_2_O. As a result, it can effectively promote the binding of key intermediate and lower the associated energy barrier. Therefore, the nickel boride/nickel heterostructure exhibits better activity for methanol oxidation than nickel boride and nickel (Supplementary Fig 14).

**Fig. 7:**
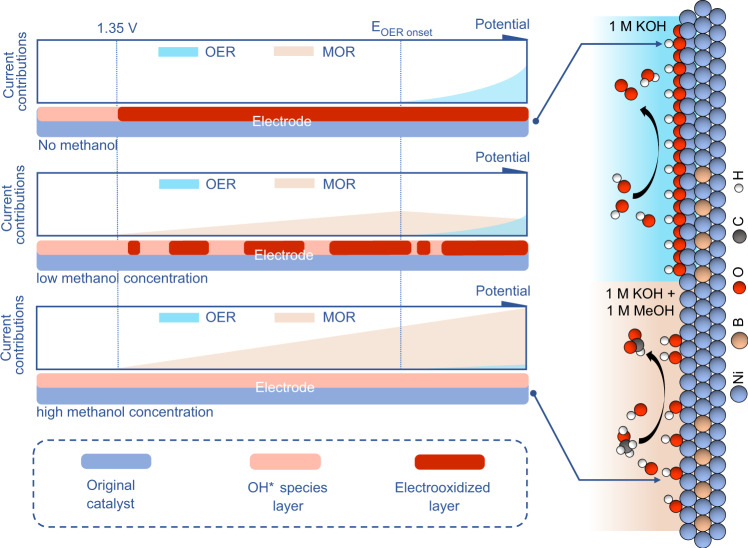
Comparison of reaction processes. Schematic representation of the methanol oxidation process on nickel boride/nickel and the evolution of the catalyst.

Taken together, the methanol oxidation process on nickel boride/nickel and the evolution of the catalyst are proposed (Fig. 7). When the methanol concentration is high, the electrooxidation of the catalyst is inhibited and the electrophilic oxygen species is the catalytically active species. In this case, MOR is most favorably occurring on the nickel boride/nickel heterostructure due to the fact that the heterstructure possesses the lowest ΔG for the potential-determining step of MOR and has the optimal adsorption for the key intermediates *CH_2_O. But when the methanol concentration is very low, the inhibitory effect of methanol on the electrooxidation of the catalyst becomes weak and the catalyst undergoes phase transition to generate high-valent products such as NiOOH. Methanol molecules are easily oxidized by the oxidative NiOOH. NiOOH is reactive to both OER and MOR, which can lead to a vicious competition. Therefore, increasing the methanol concentration can inhibit the OER.

## Discussion

In summary, we have reported a heterostructure of nickel boride/nickel catalyst for methanol electrooxidation. Only an anodic potential of about 1.51 V has been required to achieve a current density of 100 mA cm^−2^ on the optimized nickel boride/nickel catalyst. Nearly 100% formate Faradaic efficiency and good long-term stability have also been delivered. Combining electrochemical measurements, operando electrochemical impedance spectroscopy and in situ Raman spectroscopy, the effect of methanol concentration on the mechanism of MOR is presented. The formation of high-valent oxidation products of the electrocatalyst is inhibited at high methanol concentrations, and the electrophilic oxygen species (OH* or O*) is the catalytically active species for MOR. DFT calculations indicate that the heterostructure can effectively promote the binding of key intermediate and lower the associated energy barrier, and thus has superior MOR performance over nickel boride and nickel. When the methanol concentration is low, the inhibitory effect of methanol on catalyst electrooxidation decreases and methanol is easily oxidized by the high-valent electrooxidation products of the electrocatalyst. The proposed mechanism can well explain the effect of the presence of methanol on the selectivity towards the anodic reactions, catalyst passivation and catalyst electrooxidation. The understanding can readily be generalized to other nucleophile electrooxidations and organic electrosynthesis.

## Methods

### Chemicals and materials

Sodium borohydride (NaBH_4_, ≥ 98%, Weng Jiang Reagent), Nickel(II) nitrate hexahydrate ((Ni(NO_3_)_2_·6H_2_O, 99%, Acros Organics), Methanol (≥ 99.9%, Aladdin), Potassium hydroxide (KOH, 95%, Macklin), Nafion (5 wt. % in mixture of lower aliphatic alcohols and water, contains 45% water), Deuterium oxide(99.9% atom% D, contains 0.05 wt% TMSP, SIGMA-ALDRICH), Diboron trioxide (B_2_O_3_, ≥ 98%, SCR),Ethanol (≥99.7%, GENERAL-REAGENT), Tetraethylene glycol (99%, Shanghai Canspec), DI water (18.25 MΩ·cm^−1^), Nickel (20–100 nm, 99.9%, Macklin).

### Characterization

The morphology was characterized by Field-emission scanning electron microscopy (FESEM, Hitachi, S-4800) with an acceleration voltage of 15 kV. The microstructure observation was performed by Transmission electron spectroscopy (TEM, FEI F30). Phase compositions of the electrocatalysts were examined using a Rigaku Ultima IV operated under a voltage of 40 kV and a current of 40 mA. X-ray photoelectron spectroscopy (XPS) was analyzed by VG Escalab 250xi. All XPS patterns were corrected with C 1 *s* signal at 284.8 eV as the standard. Raman spectroscopic measurements were performed using a LabRAM HR800 confocal microscope (Horiba Jobin Yvon) (Excitation wavelength: 532 nm; Power: 25%; Acquisition time: 80 s). ICP was performed on Agilent 725. Attenuated Total Reflection Surface-Enhanced Infrared Absorption (ATR-SEIRA) measurements were performed on ThermoFisher Nicolet is50 (dector: MCT/A; number of scans:16; moving mirror speed: 0.4747; ink dosage: 800 μg in 80 μL). The optical crystal used here is a silicon prism, and a gold film is sputtered on its working surface using a sputter coater to enhance the signal (parameters of the sputter coater: current: 20 mA; duration: 250 s). Previous studies have shown that the effect of Au film on the MOR is negligible for the selected potential range^23^.

### Synthesis of NiB-T

Ni(NO_3_)_2_·6H_2_O (1.175 g) was dissolved completely in 100 mL of DI water. NaBH_4_ solution (0.232 g, dissolved in 10 mL DI water) was added into the solution later and stirred for 2 h. It should be noted that the reaction should be carried out in a cold water bath at around 10 °C. The precipitate was then washed three times with cold DI water (~10 °C) and cold ethanol (~10 °C) to remove impurities. All the water and ethanol used in the synthesis and washing process are ventilated with nitrogen for 15 min to remove oxygen. The obtained black precipitate was vacuum dried at 60 °C. Completely dry precipitate was named NiB_x_. NiB_x_ was divided into several parts and heated to 300, 350, 400, 450, 500 °C respectively with a heating rate of 5 °C/min and held for 3 h in H_2_/Ar (v/v = 5/95). The obtained black powders were named NiB-300, NiB-350, NiB-400, NiB-450 and NiB-500, respectively.

### Synthesis of Ni_3_B

The synthetic process of Ni_3_B referred to the reported literature with some modifications^41^. In detail, 238 mg of nickel chloride and 30 mL of Tetraethylene glycol (TEG) were added into the 150 mL Round-bottom flask. The solution was sonicated for 60 min until most of the salts were dissolved and then allowed to stir and heat to 45 °C on an oil bath pan while purging with Ar all the time. Next, 30 mL of TEG with 1 g of NaBH_4_ was added to the metal salt solution within 1 min. The color of the solution quickly turned black. The temperature was slowly warmed up to 280 °C and maintained for 5 min with continuous stirring under an Ar purge. Lastly, after being naturally cooled down, the product was collected by centrifugation with ethanol for several times.

### Synthesis of Ni

Ni(NO_3_)_2_·6H_2_O (1.175 g) was dissolved completely in 100 mL of DI water. NaBH_4_ solution (0.232 g, dissolved in 10 mL DI water) was added into the solution later and stirred for 2 hours. The reaction was carried out at around 28 °C. The precipitate was then washed three times with DI water and ethanol to remove impurities. The obtained gray precipitate was vacuum dried at 60 °C. The dried precipitate was then heated to 400 °C with a heating rate of 5 °C/min and held for 3 hours in H_2_/Ar (v/v = 5/95).

### Electrochemical measurements

The electrochemical tests were performed in a three-electrode system (25 ± 1 °C) on CHI 760E electrochemical workstation. The electrolyte was 1 M KOH aqueous solution for OER and 1 M KOH (containing 1 M methanol) for MOR if no special instruction. To prepare the working electrode, 5 mg catalysts were mixed with 460 μL ethanol and 40 μL Nafion solution with ultrasonication. 5 μL of the obtained suspension was dropped onto a φ5 mm glassy carbon electrode to be used as the working electrode. Hg/HgO (1 M KOH) and graphite rod were used as a reference electrode and a counter electrode, respectively. Unless otherwise specified, the scanning rate of LSV and CV was set as 5 mV/s. Electrochemical impedance spectroscopy (EIS) was performed over a frequency range from 0.01 Hz to 100000 Hz and AC amplitude was set as 5 mV. Electrochemical data was not adjusted by iR compensation if no special instruction is given. All potentials were calibrated to the reversible hydrogen electrode (RHE) reference scale using the formulas as follows:

1 $${{{{{{\rm{E}}}}}}}_{{{{{{\rm{RHE}}}}}}}={{{{{{\rm{E}}}}}}}_{{{{{{\rm{Hg}}}}}}/{{{{{\rm{HgO}}}}}}}+0.0591\times {{{{{\rm{pH}}}}}}+0.098$$

2 $${{{{{{\rm{E}}}}}}}_{{{{{{\rm{RHE}}}}}}}={{{{{{\rm{E}}}}}}}_{{{{{{\rm{Ag}}}}}}/{{{{{\rm{AgCl}}}}}}}+0.0591\times {{{{{\rm{pH}}}}}}+0.197$$

### The calculation of electrochemical double-layer capacitance

Electrochemical double-layer capacitance (C_dl_) was measured by cyclic voltammetry. The potential window was selected in a range of OCP ± 50 mV. The scan speed was set to 5, 10, 15, 20, 25, 30, 35, 40, 45 mV/s. The Δj at the open circuit potential was plotted against the scan rate and the slope fitted was the estimated value of C_dl_.

### EIS fitting

For the OER process, the equivalent circuit before 1.35 V consists of a solution resistor (R_s_) in series with R_1_ | | CPE_1_, which reflects the electrooxidation of the catalyst. After 1.35 V, the equivalent circuit consists of a solution resistor, an R_1_ | | CPE_1_ and R_2_ | | CPE_2_ in series. A new R_2_ | | CPE_2_ appears to reflect the OER process, and R_2_ is the charge transfer resistance of the OER. For the MOR process (1 M KOH + 1 M methanol), since the electrooxidation of the catalyst is suppressed, the equivalent circuit is composed of a solution resistance (R_s_) and R_1_ | | CPE_1_ in series, and R_1_ | | CPE_1_ reflects the MOR process.

Often a CPE is used in a model in place of a capacitor to compensate for non-homogeneity in the system. The CPE is defined by two values, CPE-T and CPE-P. If CPE-P equals 1, then CPE is equivalent to a capacitor. The contributions of the capacitive resistances of the EIS results for the OER and MOR processes were fitted according to the above circuits. The fitting results are detailed in Supplementary Table 3, 4.

### Product analysis

Nuclear magnetic resonance (Ascend 600) was used to analyze the product content of the methanol oxidation reaction. First, a series of potassium formate (HCOOK) solutions of different concentrations (1 mM, 3 mM, 5 mM, 7 mM, 10 mM, 15 mM, 20 mM) were prepared. 500 μL of potassium formate solution and 100 μL of D_2_O (containing 0.05 wt% TMSP) were added into a NMR tube and mixed thoroughly for NMR test. A standard curve was drawn based on the proportional relationship between the integrated intensity of the formate ion signal peak and the concentration of formate ion. Typically, for the analysis of the product in the electrolyte, 500 μL of electrolyte and 100 μL of D_2_O (containing 0.05 wt% TMSP) were added in a NMR tube and mix them thoroughly. Then the nuclear magnetic resonance test was performed to obtain the integrated intensity of the formate ion signal. The content of formate produced was calculated according to the standard curve.

### DFT calculations

Geometry optimization and electronic structure computation have been carried out by using the Vienna abinitio simulation package (VASP)^42^. The projector-augmented-wave (PAW) method together with the generalized gradient approximation-Perdew-Burke-Ernzerhof (GGA-PBE) functional were employed in the simulation process^43^. An energy cutoff of 450 eV was used for the plane-wave expansion of the electronic wave function. All geometric structures were relaxed until the energy and forces were converged to 10^−5^ eV and −0.02 eV Å^−1^, respectively. A system of 2 × 2 slab with 4 layers was employed to model the Ni surfaces and a 2 × 2 slab with 1 layer was used to simulate the Ni_3_B surface. A vacuum layer of 15 Å along the z-direction was applied to separate the surfaces. The geometric model of Ni (111) and Ni_3_B (001) were chosen to demonstrate the pristine Ni and Ni_3_B materials. To simulate the Ni_3_B/Ni heterostructure, we built the layered model to investigate the interaction effect between Ni and Ni_3_B, as well as the complete mechanism of MOR on such hybrid catalyst. The Brillouin zone was sampled with 3 × 2 × 1 Monkhorst-Pack k-meshes for the geometry optimization, while 6 × 4 × 2 grid of K-points were used for the more accurate electronic states analysis.

The Gibbs free energy change (ΔG) of each reaction step was calculated according to the following formula:

3 $$\Delta {{{{{\rm{G}}}}}}=\Delta {{{{{{\rm{E}}}}}}}_{{{{{{\rm{ads}}}}}}}+\Delta {{{{{{\rm{E}}}}}}}_{{{{{{\rm{ZPE}}}}}}}{{{{{\rm{\hbox{-}}}}}}}{{{{{\rm{T}}}}}}\Delta {{{{{\rm{S}}}}}}$$

where ΔE_ads_ is the adsorption energy of reaction intermediates; ΔE_ZPE_ and ΔS are the energy difference in zero point energy and entropy, respectively. Therein, a more negative E_ads_ implies that the adsorption is thermodynamically more favorable.

## Supplementary information

Supplementary Information

## Data Availability

The data that support the findings of this study are available from the corresponding author upon reasonable request. Source data are provided with this paper.

## Source data

Source Data
