# Supplementary material for: Insights into the activity of nickel boride/nickel heterostructures for efficient methanol electrooxidation
Source: Nat Commun. 2022 Aug 6;13:4602. doi: 10.1038/s41467-022-32443-5 (PMC9357015; doi:10.1038/s41467-022-32443-5)
Supplement: Supplementary file 1 — Supplementary Information [file 41467_2022_32443_MOESM1_ESM.pdf]

Insights into the activity of nickel boride/nickel heterostructures for efficient methanol electrooxidation

Yanbin Qi[1,2], Yue Zhang[3], Li Yang[3]*, Yuhan Zhao[2], Yihua Zhu[2], Hongliang Jiang[1]*, and Chunzhong Li[1,2]*

[1]Key Laboratory for Ultrafine Materials of Ministry of Education, School of Chemical Engineering, East China University of Science and Technology, Shanghai 200237, China.

[2]Shanghai Engineering Research Center of Hierarchical Nanomaterials, School of Materials Science and Engineering, East China University of Science and Technology, Shanghai 200237, China.

[3]Institutes of Physical Science and Information Technology, Anhui University, Hefei 230601, China.

These authors contributed equally: Yanbin Qi, Yue Zhang.

*Corresponding author

E-mail address: lyang@ahu.edu.cn (Li Yang); jhlworld@ecust.edu.cn (Hongliang Jiang); czli@ecust.edu.cn (Chunzhong Li).

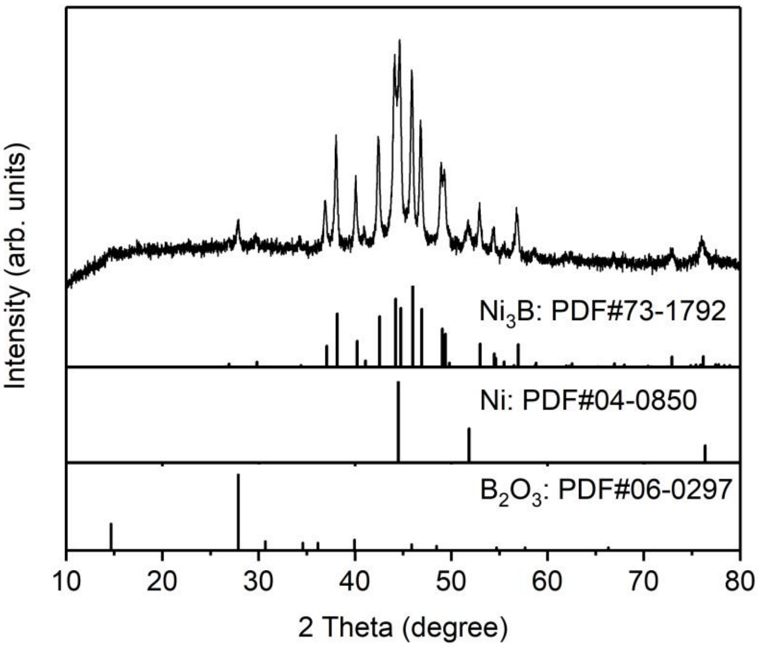

Supplementary Fig. 1. Phase characterization. XRD pattern of NiB-400.

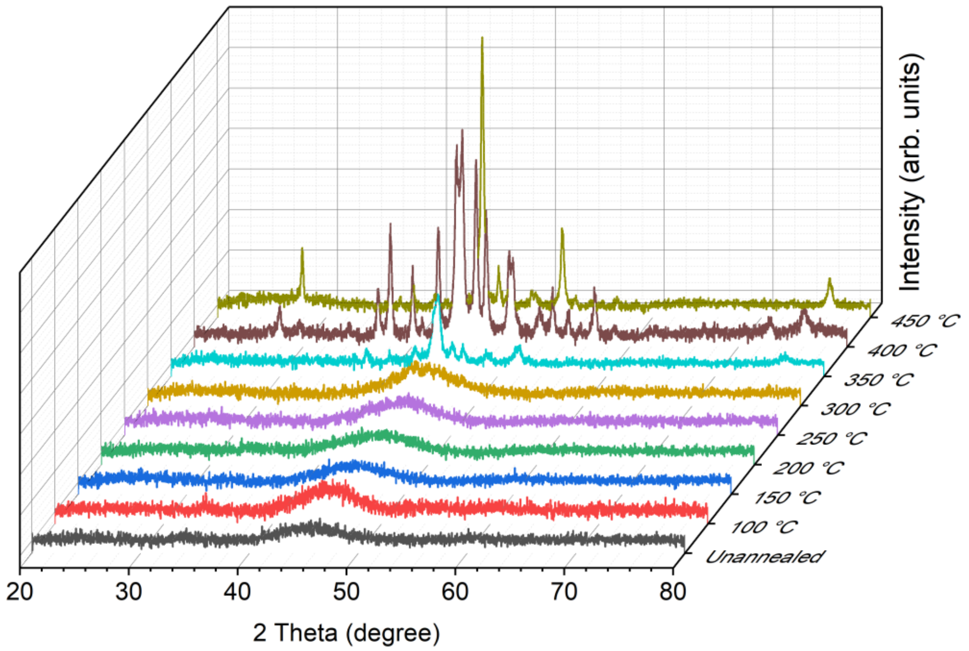

Supplementary Fig. 2. The influence of annealing temperature on the phase structure. XRD patterns of the samples annealed at different temperature.

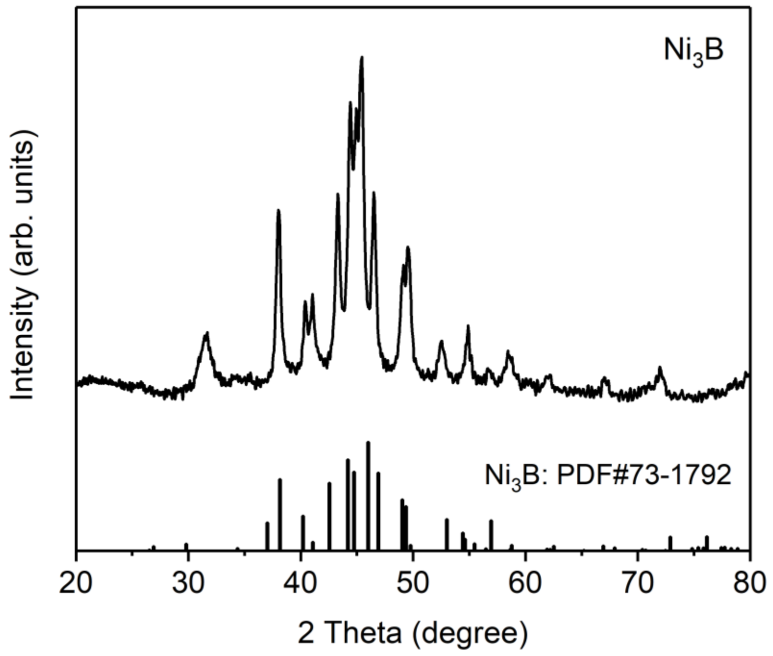

Supplementary Fig. 3. Phase characterization. XRD pattern of as prepared Ni_3B.

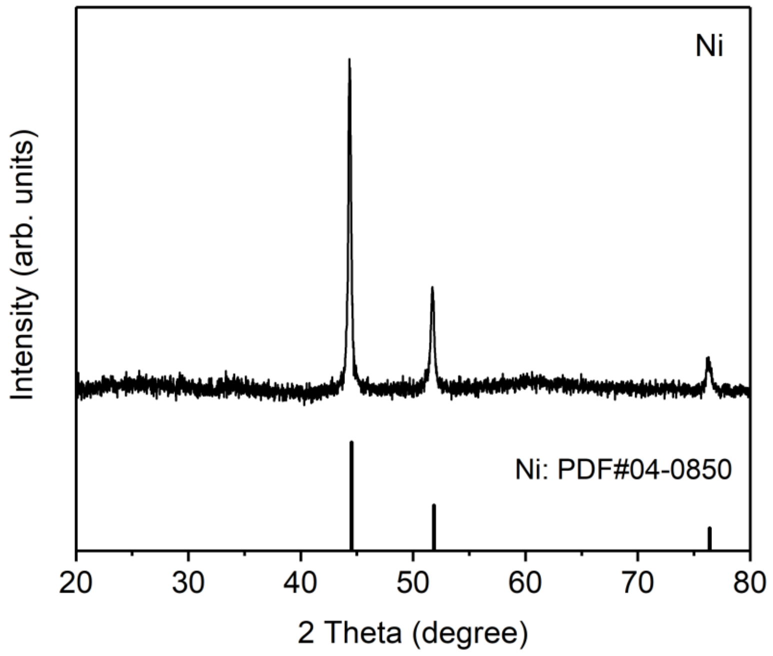

Supplementary Fig. 4. Phase characterization. XRD pattern of as prepared Ni.

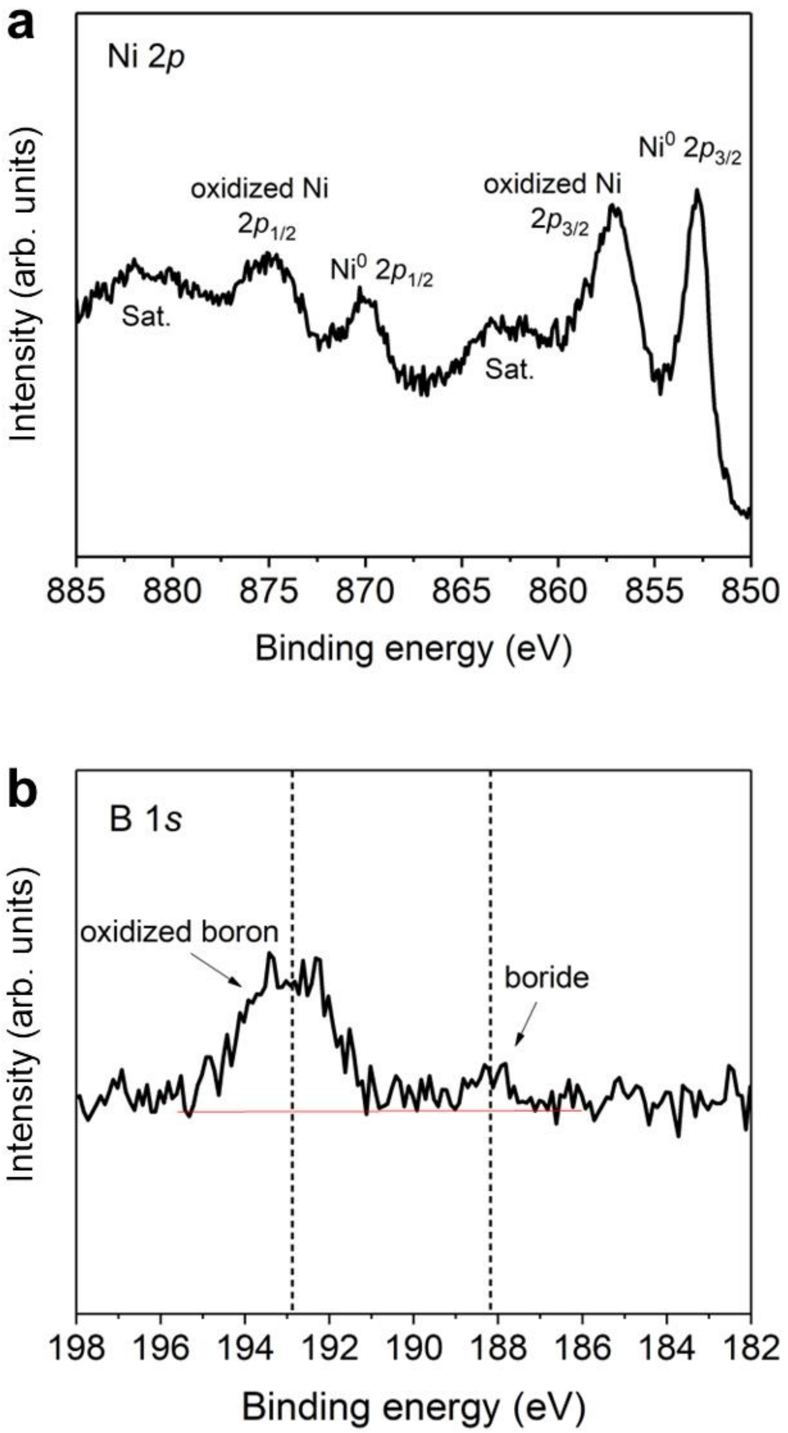

Supplementary Fig. 5. Surface analysis. High resolution **a** Ni 2*p* and **b** B 1*s* XPS spectra of NiB-400.

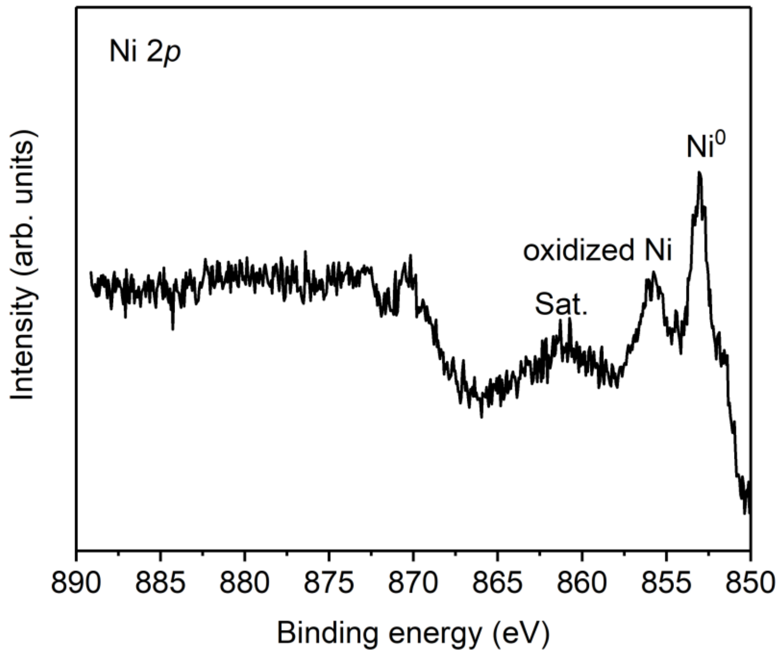

Supplementary Fig. 6. Surface analysis. High resolution Ni 2*p* XPS spectrum of Ni_3B.

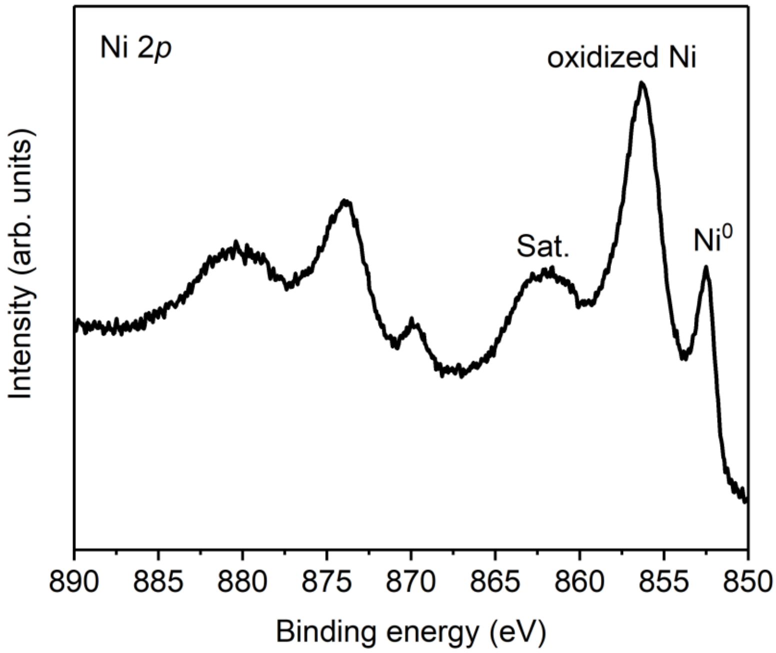

Supplementary Fig. 7. Surface analysis. High resolution Ni 2*p* XPS spectrum of Ni.

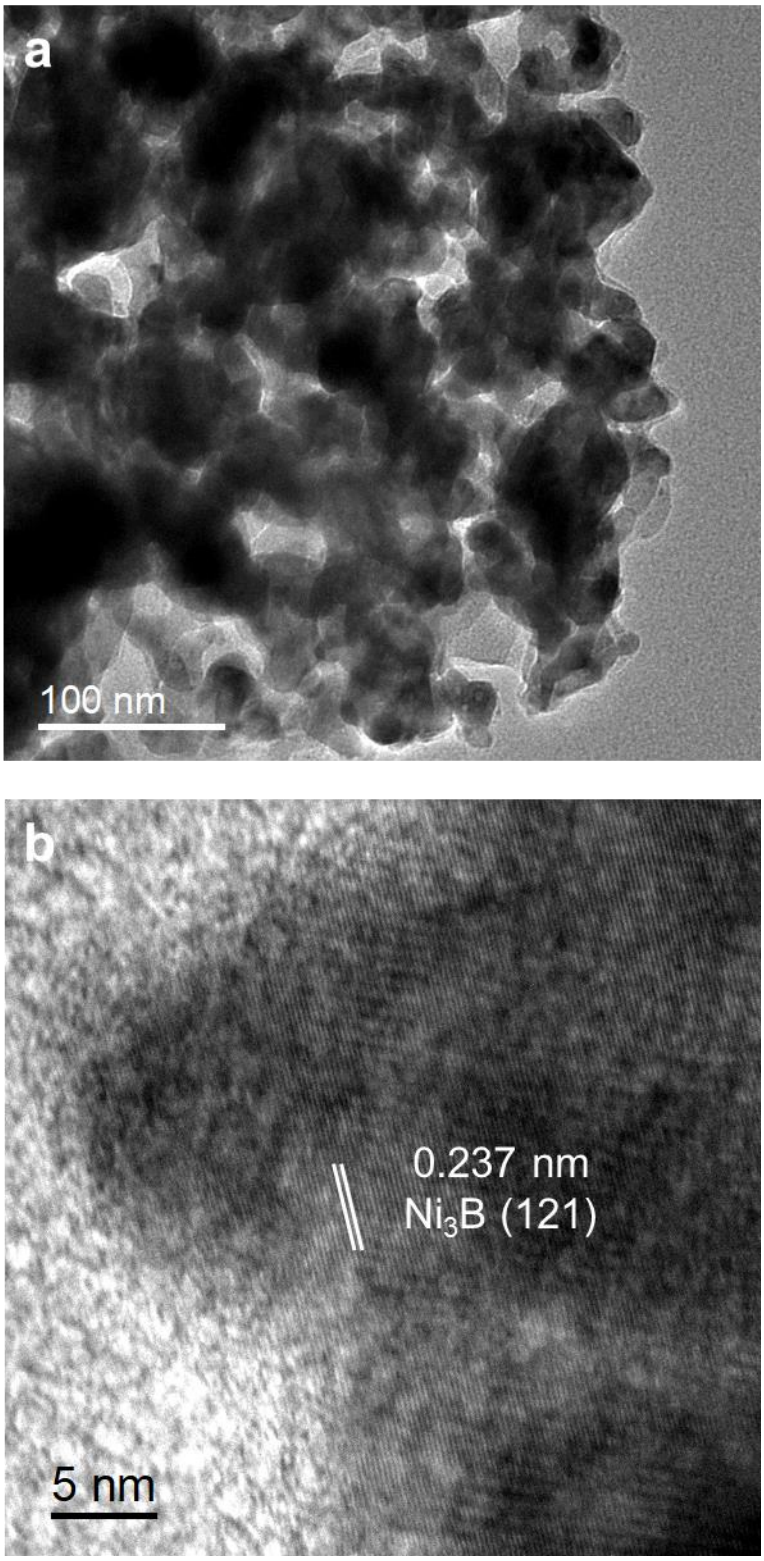

Supplementary Fig. 8. Microstructure characterization. a TEM image and **b** HRTEM micrograph of the prepared Ni_3B.

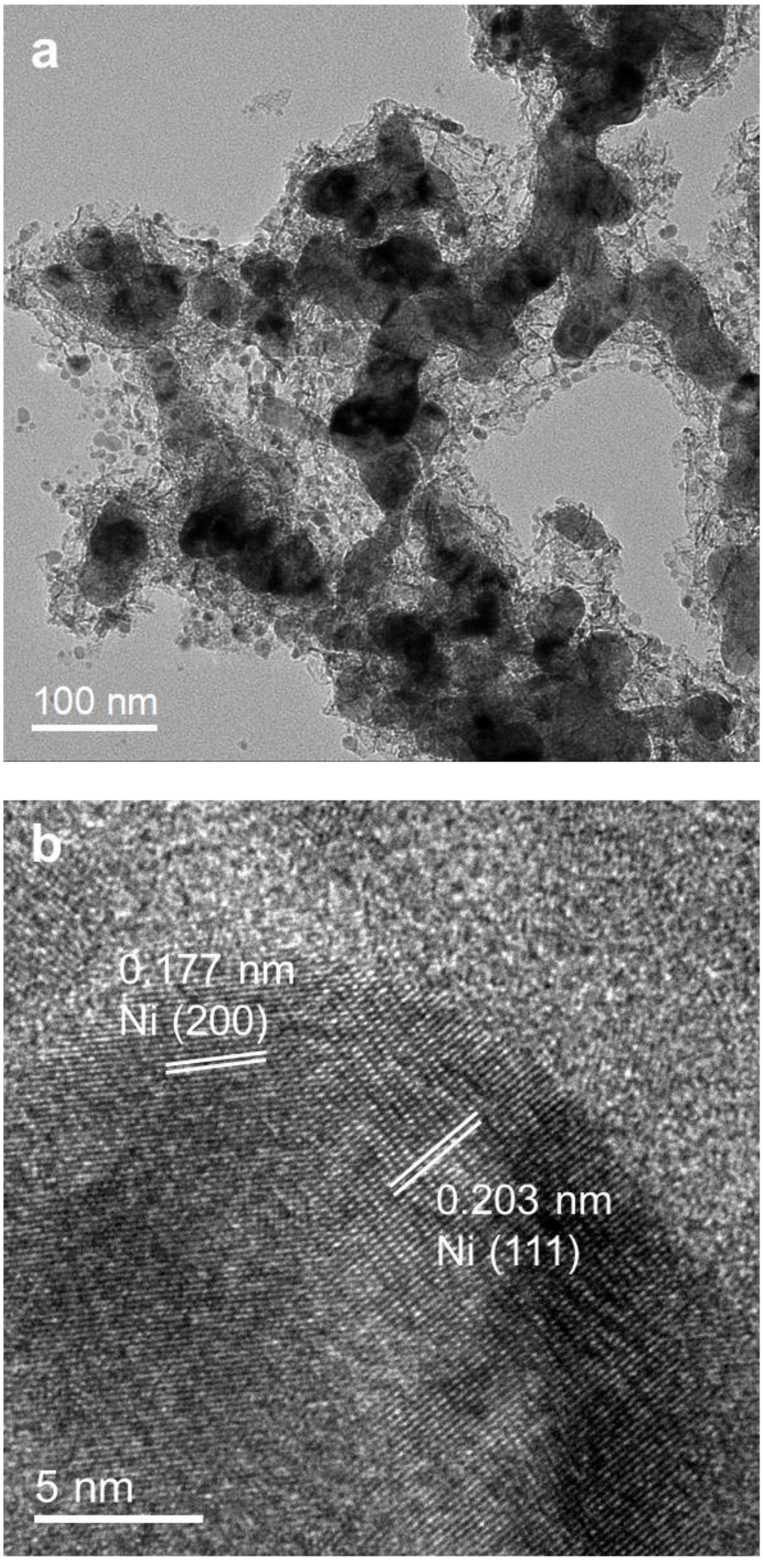

Supplementary Fig. 9. Microstructure characterization. a TEM image and **b** HRTEM micrograph of the prepared Ni.

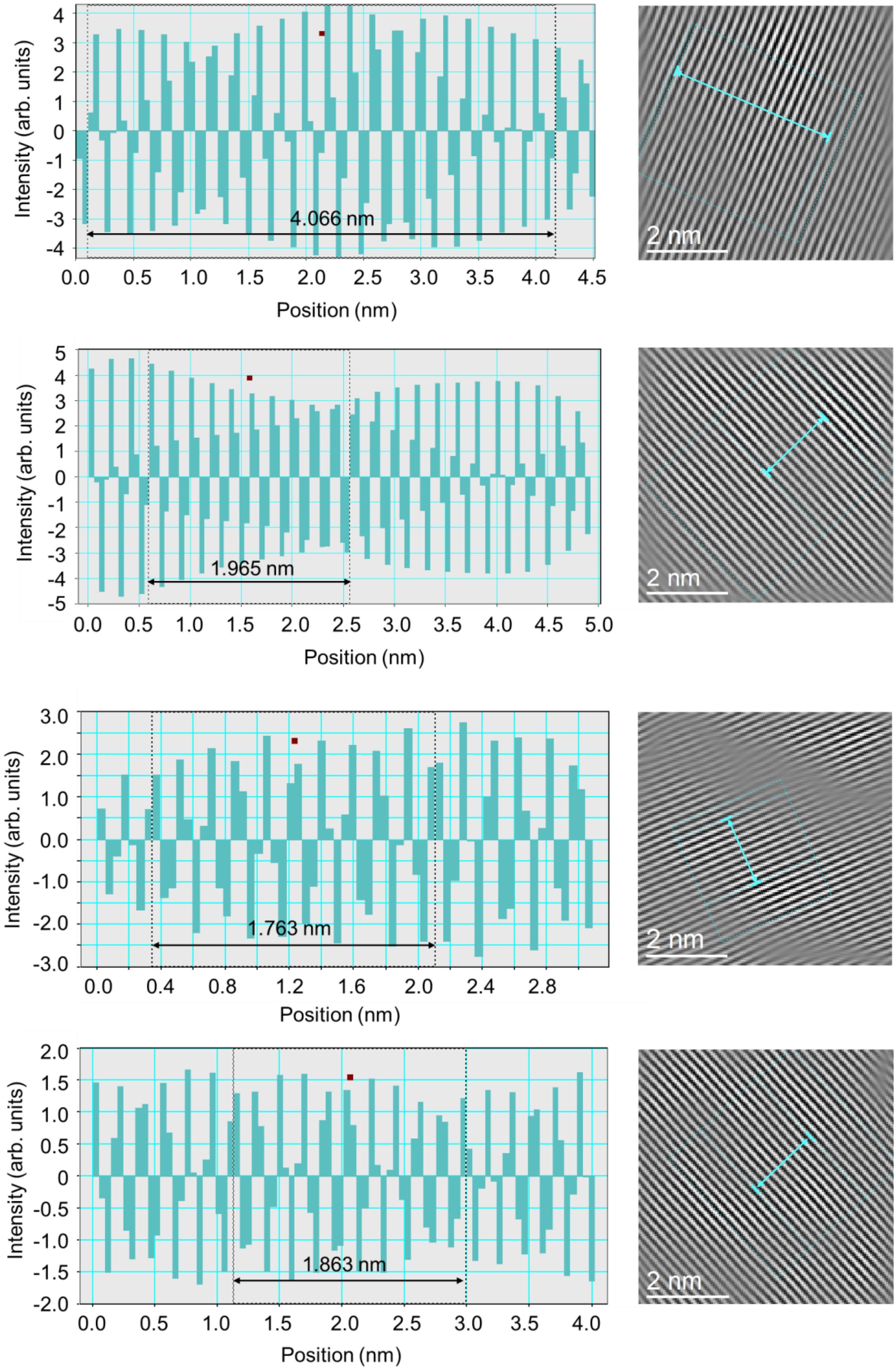

Supplementary Fig. 10. Measurement of lattice spacing. Intensity profiles measured from the regions in Fig. 1f-i.

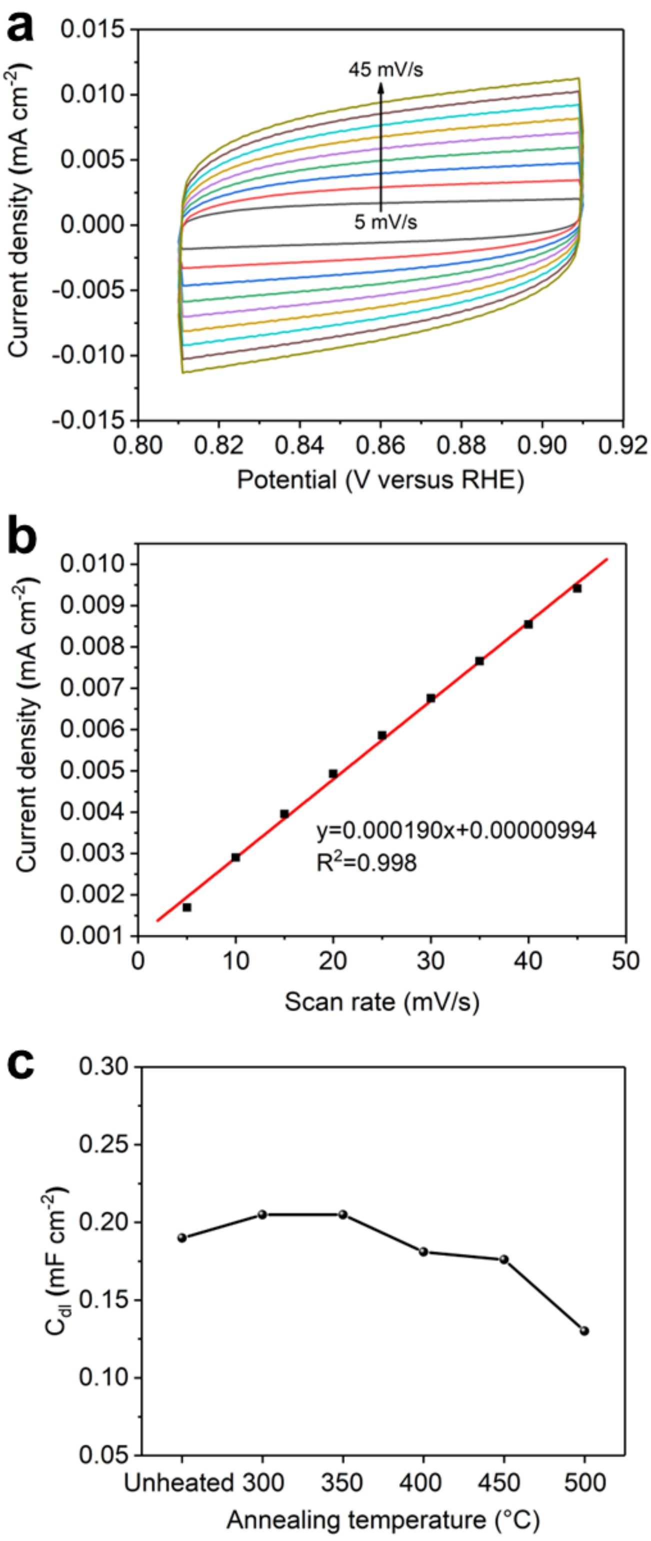

Supplementary Fig. 11. Determination of double layer capacitance. **a** Cyclic voltammetry curves for NiB_x performed at different scan rates. **b** Double-layer charging current plotted against the CV scan rate for NiB_x. **c** C_{dl} of samples annealed at different temperatures.

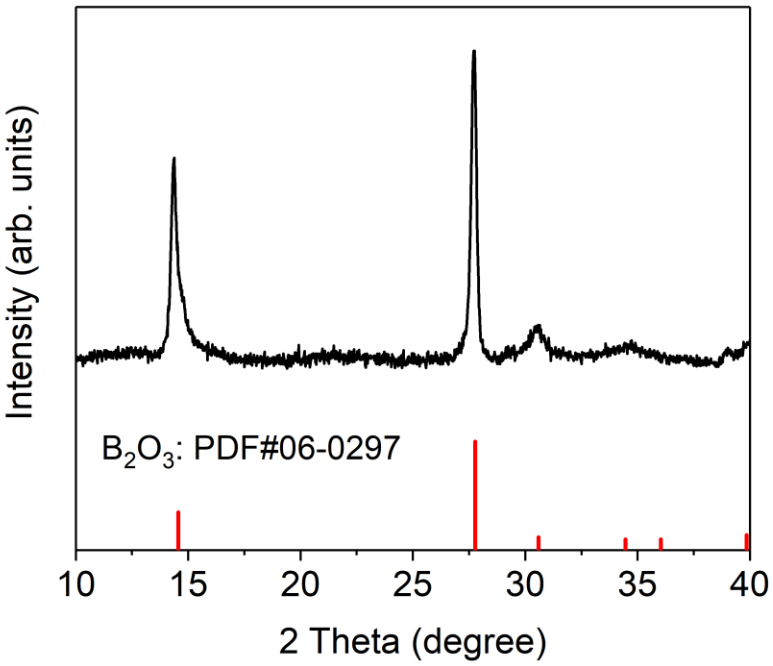

Supplementary Fig. 12. Phase characterization. XRD pattern of pure B_2O_3.

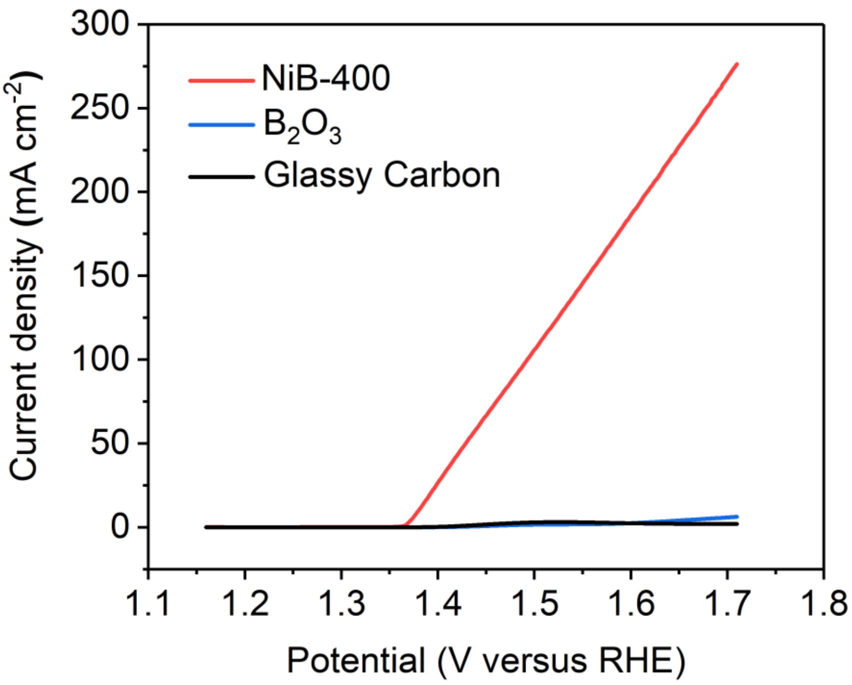

Supplementary Fig. 13. Performance of substrate and impurity. Performance comparison of pure B_2O_3, clean glassy carbon and NiB-400 (in 1 M KOH + 1 M MeOH).

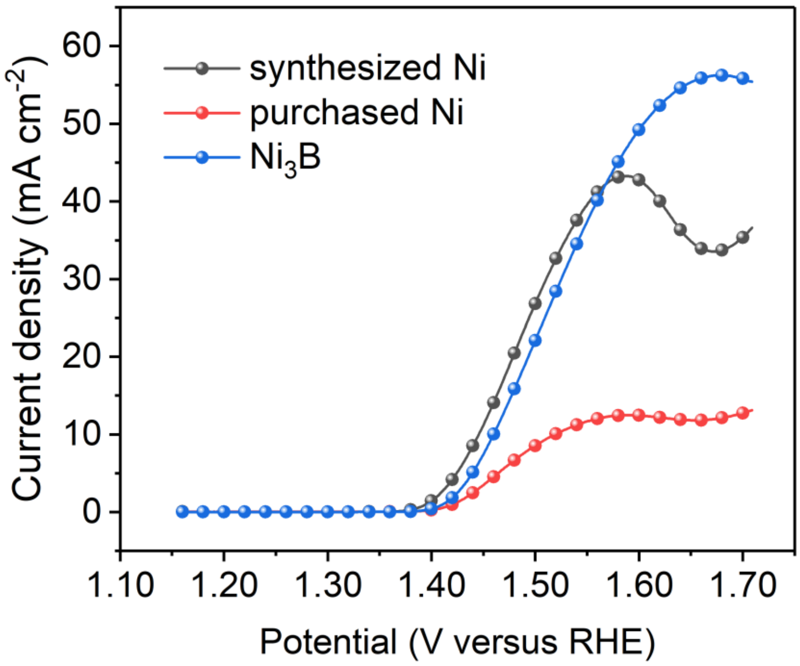

Supplementary Fig. 14. Performance of each component. Performance comparison of prepared Ni_3B, prepared Ni and purchased Ni NPs (in 1 M KOH + 1 M MeOH).

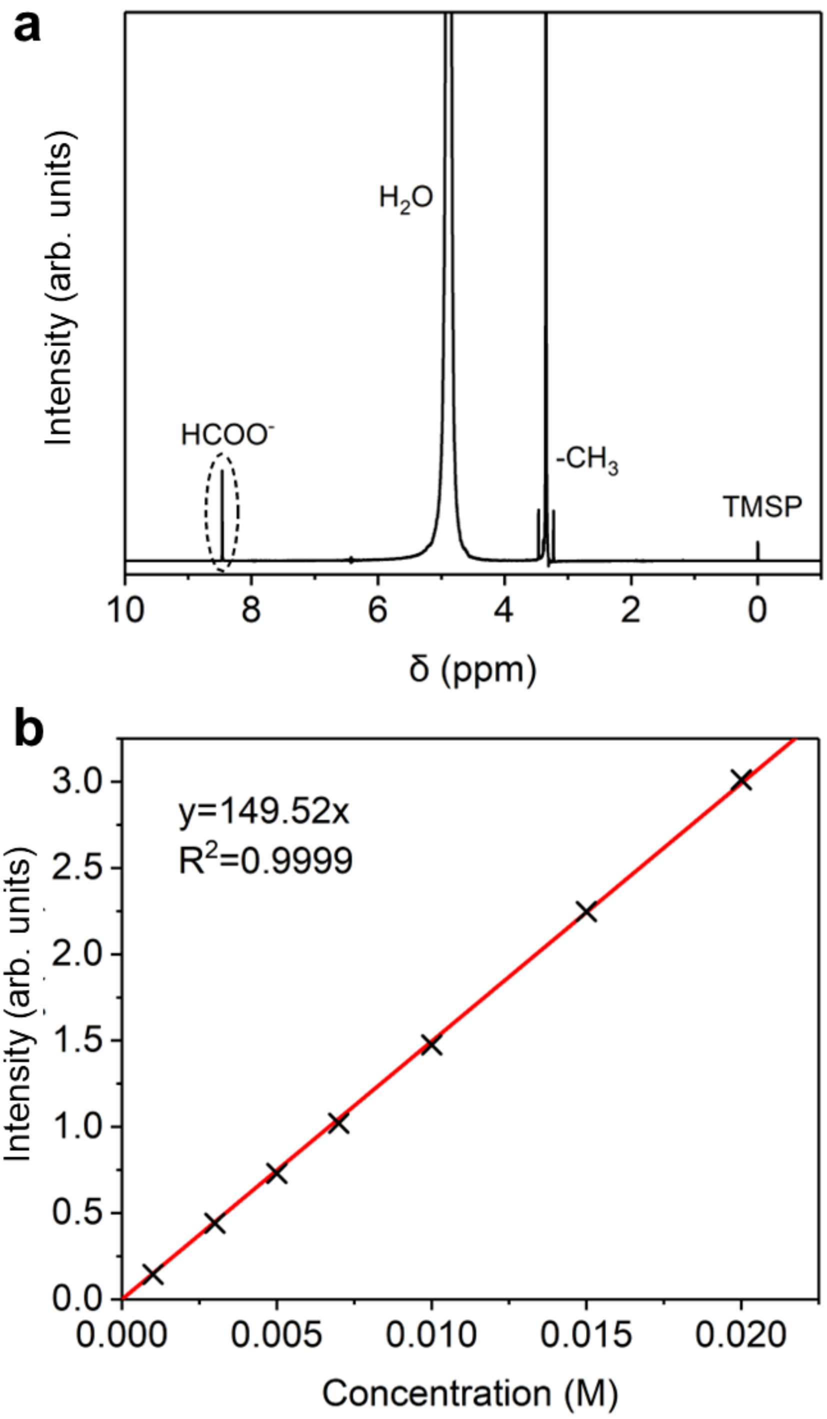

Supplementary Fig. 15. Quantification of formate. a ^{1}H NMR spectrum of the electrolyte after MOR. **b** Standard curve for formate quantification.

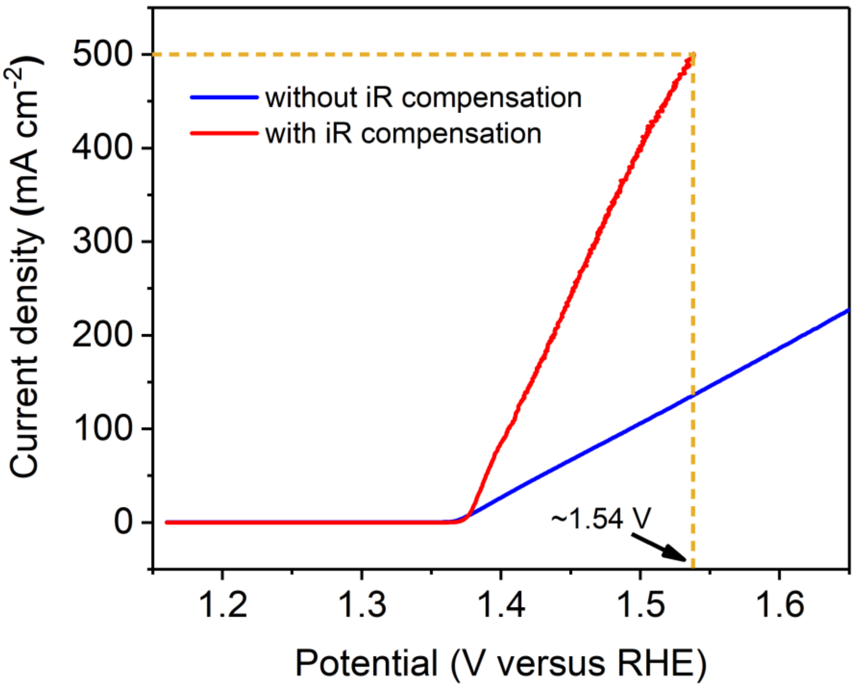

Supplementary Fig. 16. Effect of ohmic drop. LSV curve of NiB-400 before and after 95% iR compensation.

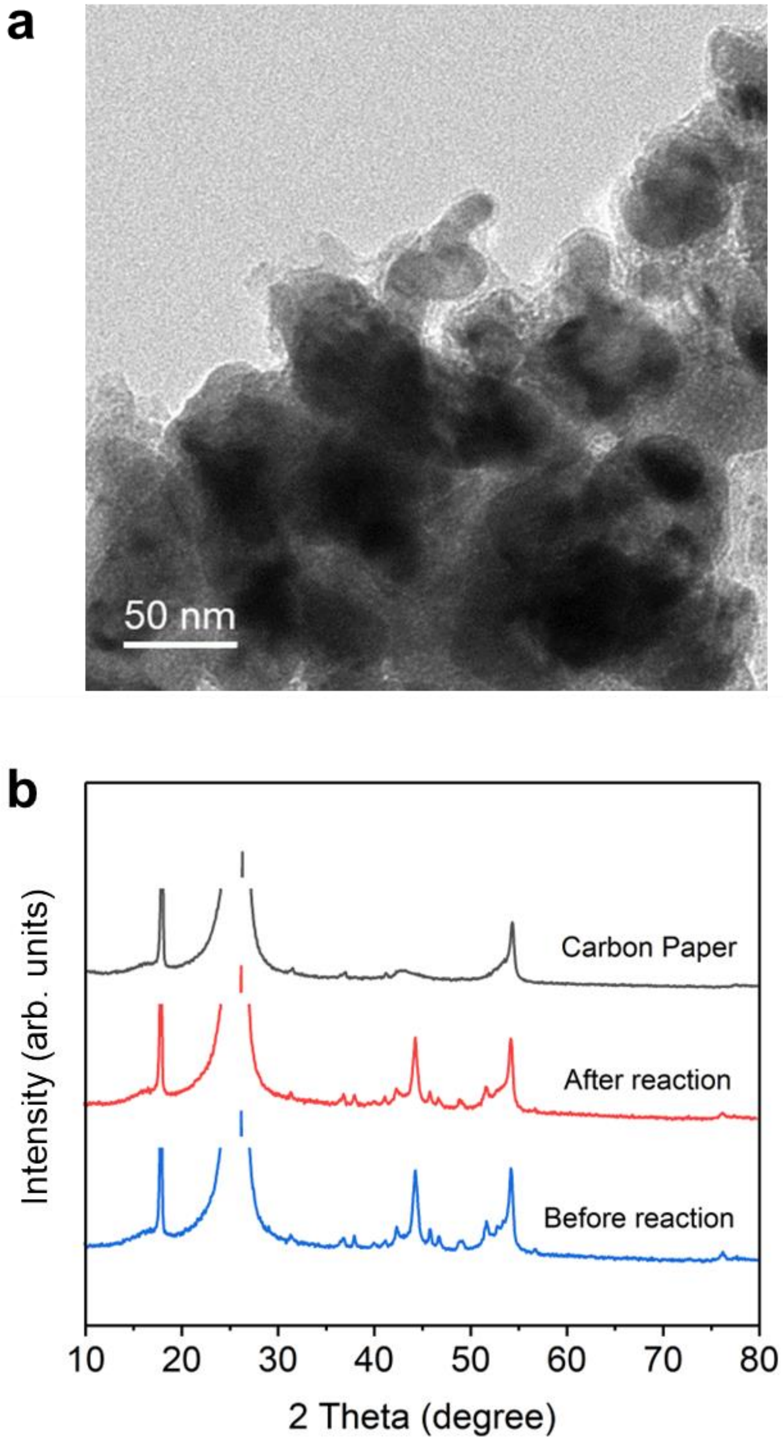

Supplementary Fig. 17. Morphology and phase after MOR. a TEM image of NiB-400 after MOR. **b** XRD patterns of clean carbon paper and NiB-400 catalyst (coated on carbon paper) before MOR and after 600 s MOR.

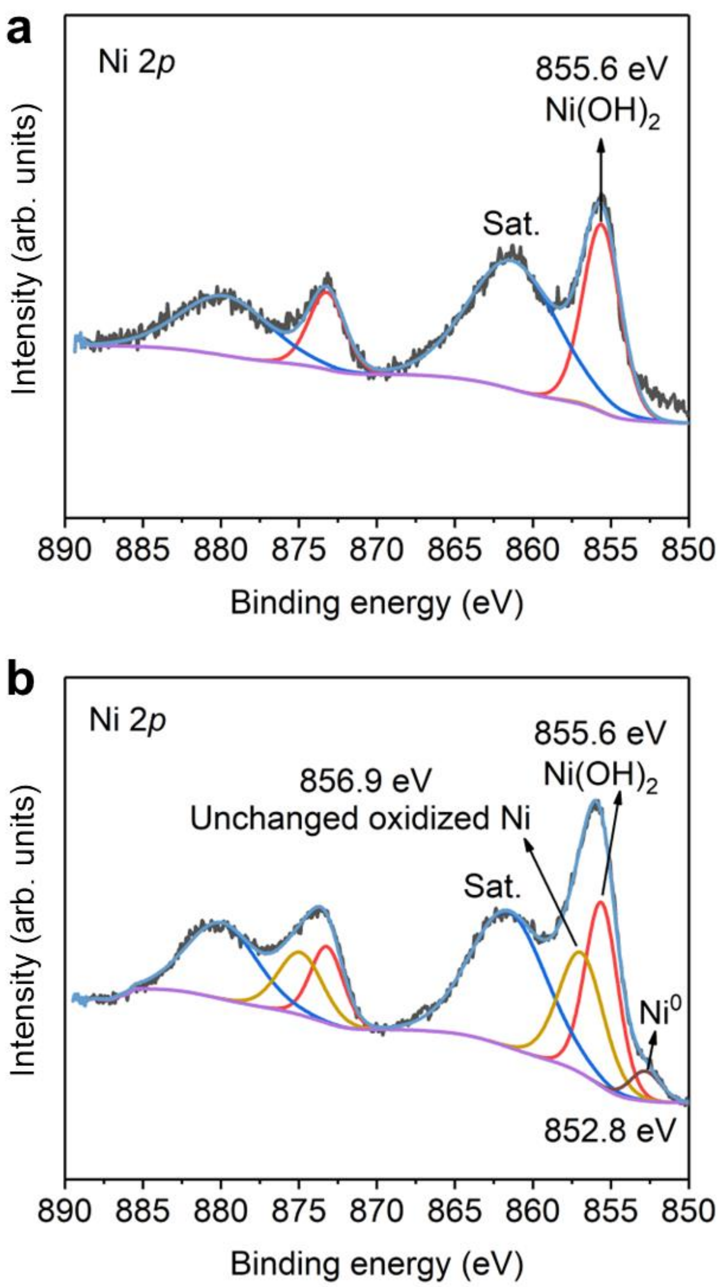

Supplementary Fig. 18. XPS spectra after reactions. High resolution Ni 2*p* XPS spectra of NiB-400 after **a** 600s OER and **b** 600 s MOR. After the OER, nickel hydroxide is formed on the surface of NiB-400. This is due to the formation of NiOOH by catalyst electrooxidation, which is reduced to nickel hydroxide after participating in the OER. After the MOR, the original surface composition of NiB-400 is retained and nickel hydroxide type-Ni appears. The formation of nickel hydroxide type-Ni is due to the formation of OH layer on the surface of the metallic nickel-based material in the alkaline electrolyte.

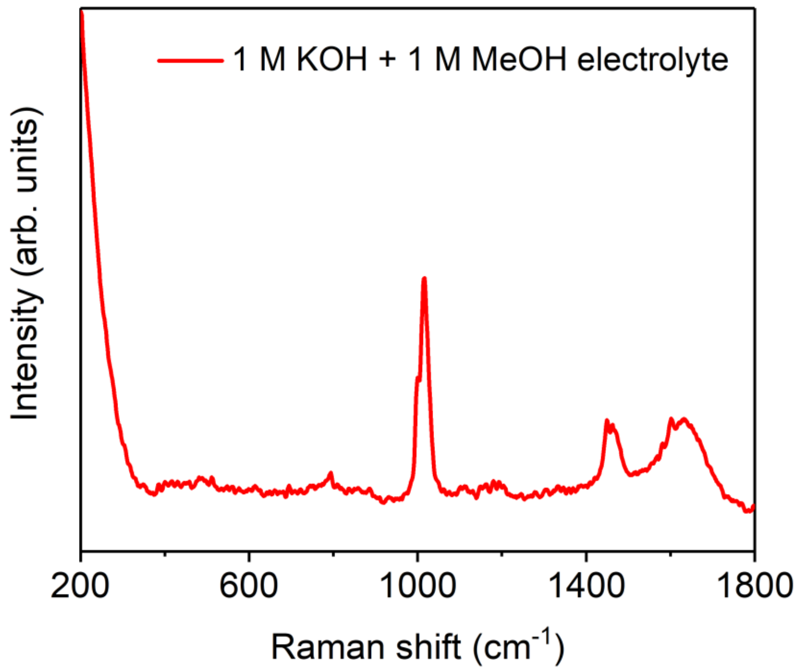

Supplementary Fig. 19. Spectral characterization of the electrolyte. Ex situ Raman spectrum of 1 M KOH + 1 M MeOH Electrolyte.

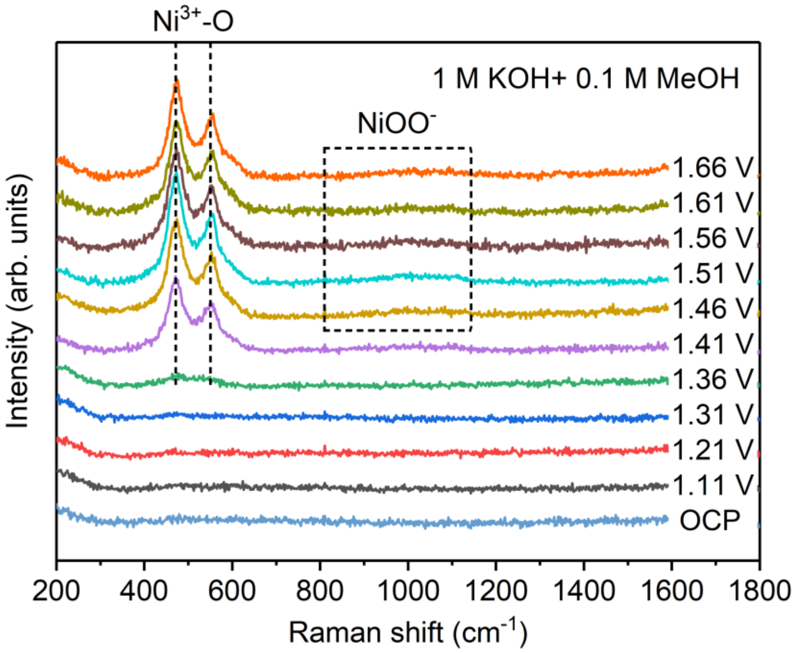

Supplementary Fig. 20. In situ Raman spectra at low methanol concentration. In situ Raman spectra measured in 1 M KOH + 0.1 M MeOH electrolyte.

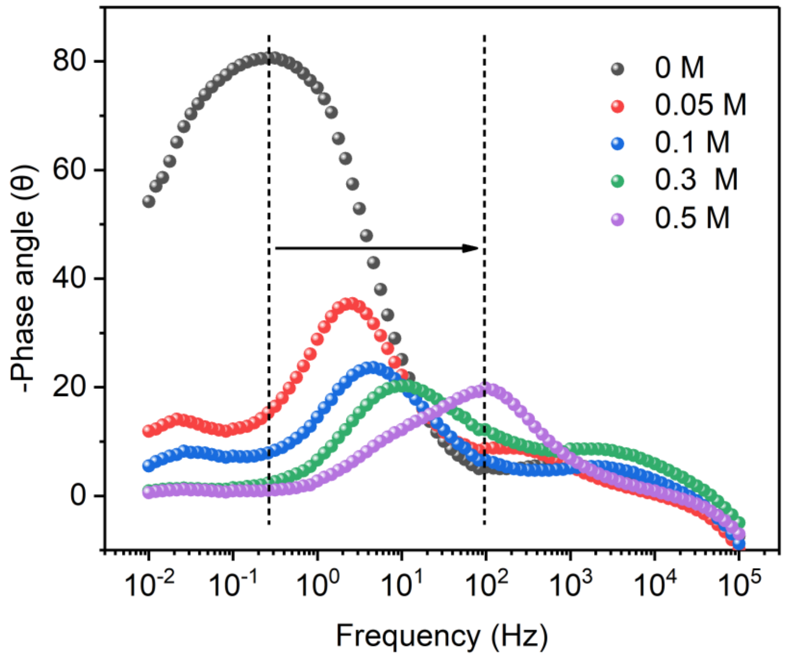

Supplementary Fig. 21. Effect of methanol concentration. Bode plots measured in electrolytes containing different concentrations of methanol at 1.41 V.

.

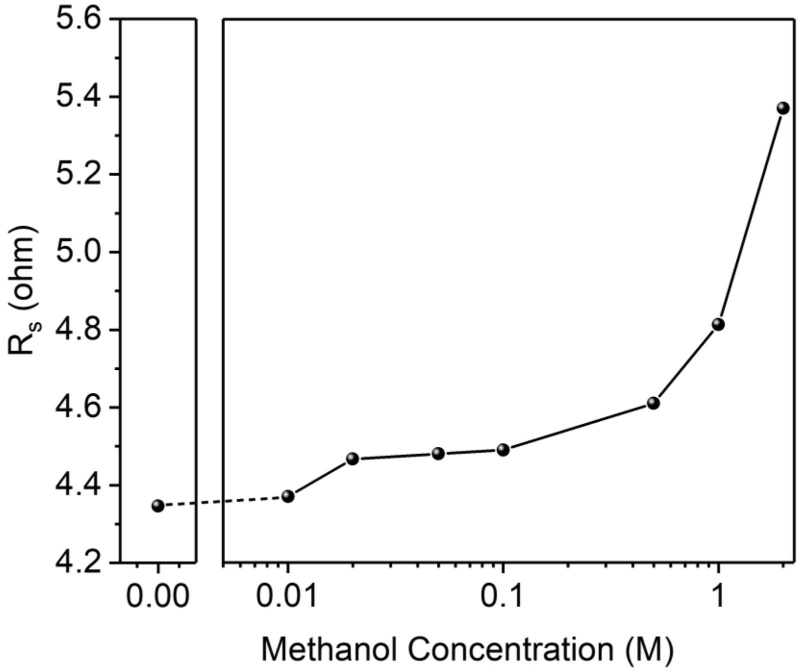

Supplementary Fig. 22. Change in solution resistance. The relationship between solution resistance and methanol concentration.

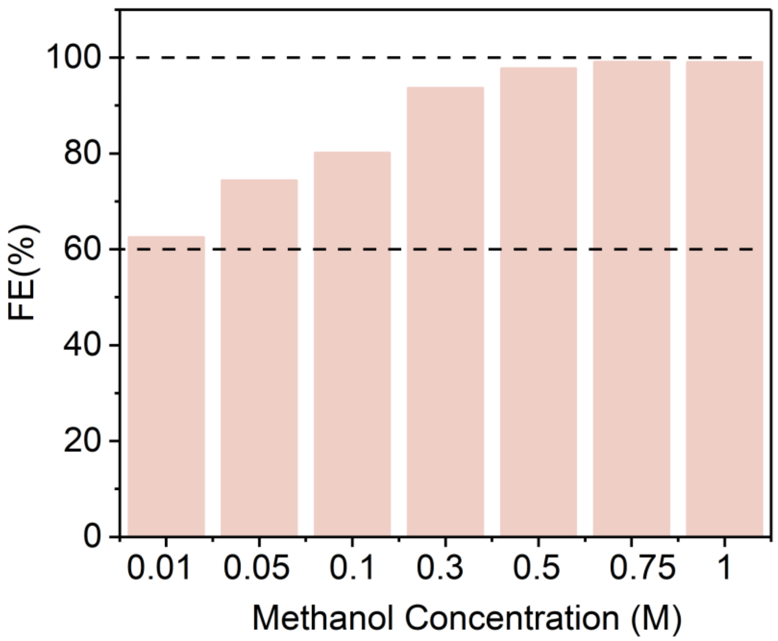

Supplementary Fig. 23. Effect of methanol concentration. Faradaic efficiency of formate after 1 h electrolysis at 1.61 V in electrolytes containing different concentrations of methanol.

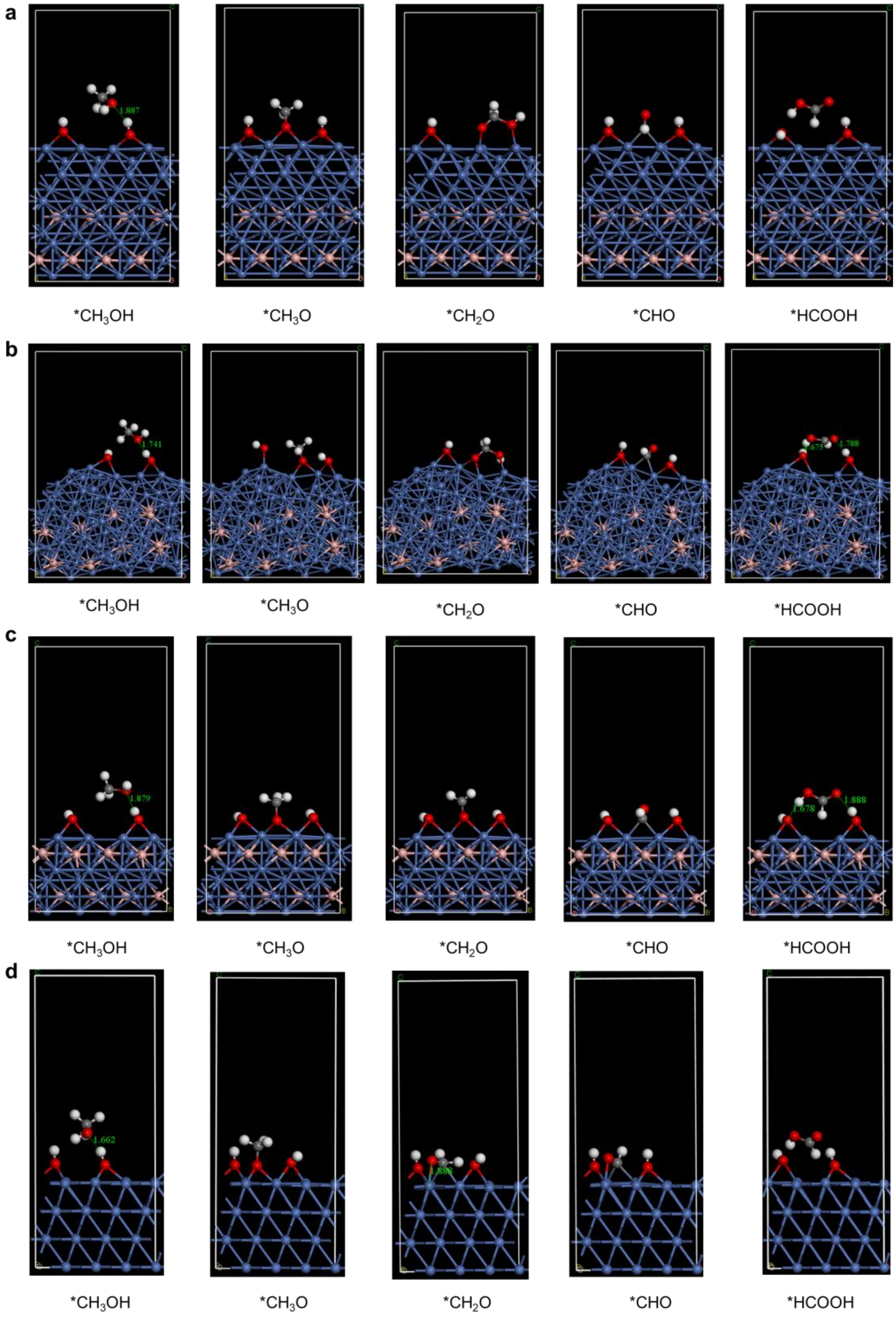

Supplementary Fig. 24. DFT calculations. DFT-optimized models of the adsorbed intermediates for the oxidation of methanol on **a** $Ni_3B(001)/Ni(111)$, **b** $Ni_3B(221)/Ni(111)$ **c** $Ni_3B(001)$ and **d** Ni(111).

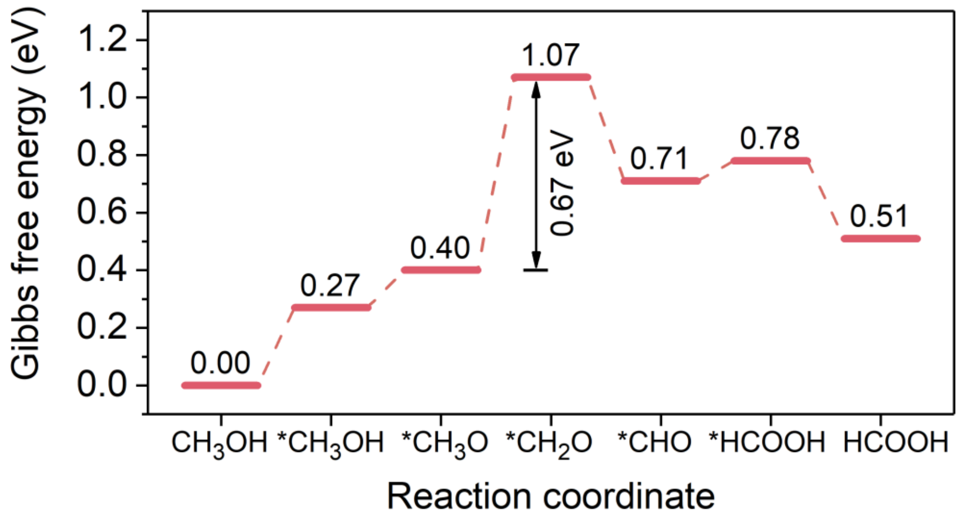

Supplementary Fig. 25. DFT calculations. Gibbs free energy diagram of MOR occurring on $Ni_3B(221)/Ni(111)$ heterostructure. On the $Ni_3B(221)/Ni(111)$ heterostructure, the potential-determining step of MOR is also $*CH_3O \rightarrow *CH_2O$. The change in Gibbs free energy for the potential-determining step is 0.67 eV, which is lower than that of Ni.

Supplementary Table 1. ICP results of NiB_x.

Element	Instrument reading	Mass content
Ni	17.450 mg/L	881328.3 mg/kg
B	1.2568 mg/L	63474.7 mg/kg

Supplementary Table 2. Ni-based electrocatalysts reported in recent years and their MOR performance in alkaline media.

Electrocatalyst	Type of electrode	Electrolyte	MOR performance			Reference
			Applied potential /V	Current density /mA cm^{-2}	iR correction	
Ni_3C NPs	Glassy carbon	1 M KOH + 1 M MeOH	1.61	127	N	1
Ni polyhedral NCs	Glassy carbon	1 M KOH +1 M MeOH	1.61	~115	N	2
Cu-Ni-S	Carbon cloth	1 M KOH + 1 M MeOH	1.56	~67	N	3
Ni/CN	Glassy carbon	1 M NaOH + 1 M MeOH	1.61	~6.3 (0.033 A mg^{-1})	N	4
NiCo/NiO-CoO/ nanoporous carbon composite	Glassy carbon	0.5 M NaOH + 0.5 M MeOH	1.61	~142	N	5
α-$Ni(OH)_2$-Ni_3S_4	Glassy carbon	1 M KOH + 0.5 M MeOH	1.61	100	N	6
Cu-Ni/CuO-NiO	Glassy carbon	1 M KOH + 0.5 M MeOH	1.7	152	N	7
$Ni_{97}Bi_3$ aerogel	Glassy carbon	1 M KOH + 1 M MeOH	1.66	200	N	8
Ni/WC hybrid NPs	Glassy carbon	1 M KOH + 1 M MeOH	1.61	325	Y	9

$Ni_{0.6}Co_{0.4}$	Glassy carbon	1 M NaOH + 0.5 M MeOH	1.61	96	N	10
Ni/N–C composite	Carbon cloth	1 M KOH + 1 M MeOH	1.66	147	N	11
Ni-NPs/ZrO_2–PCs/rGO	Glassy carbon	1 M NaOH + 0.5 M MeOH	1.6	112	N	12
Ni–Co hydroxide nanoarrays	Foam	1 M KOH + 0.5 M MeOH	1.4	~262	Y	13
$Ni(OH)_2$ nanosheet arrays	Foam	1 M KOH + 0.5 M MeOH	1.36	100	Y	14
3D porous nickel films	Film	1 M NaOH + 1 M MeOH	1.61	117	N	15
Nickel boride/nickel heterostructure	Glassy carbon	1 M KOH + 1 M MeOH	1.61	175	N	This work
Nickel boride/nickel heterostructure	Glassy carbon	1 M KOH + 1 M MeOH	1.54	500	Y	This work

Supplementary Table 3. Fitting results of EIS for OER process.

Potential (V)	R_s (Ω)	R_1 (Ω)	CPE_1-T ($S\cdot s^n\cdot cm^{-2}$)	CPE_1-P	R_2 (Ω)	CPE_2-T ($S\cdot s^n\cdot cm^{-2}$)	CPE_2-P
1.11	4.57	24875	3.17E-05	0.91187			
1.21	4.402	25782	7.24E-05	0.82801			
1.31	4.462	20157	0.00017093	0.76339			
1.36	4.619	5.965	0.00066208	0.71129	4920	0.0062698	0.91522
1.41	4.553	1.267	0.00058241	0.85603	2601	0.0066036	0.94662
1.46	4.507	0.95692	0.0019463	0.73733	2605	0.0066193	0.95631
1.51	4.498	0.92573	0.0046434	0.62791	533.2	0.004778	0.96247
1.56	4.549	0.90879	0.012489	0.52343	99.33	0.0034335	0.97142
1.61	4.826	0.79239	0.019198	0.49357	20.45	0.0029416	0.97533

Note: n=CPE-P.

Supplementary Table 4. Fitting results of EIS for MOR process.

Potential (V)	R_s (Ω)	R_1 (Ω)	CPE_1-T ($S \cdot s^n \cdot cm^{-2}$)	CPE_1-P
1.11	4.907	28250	2.94E-05	0.91888
1.21	4.664	25198	6.57E-05	0.83159
1.31	4.778	13972	0.000147	0.77165
1.36	4.973	1324	0.000394	0.70583
1.41	5.051	27.78	0.000692	0.76844
1.46	5.103	12.31	0.000389	0.87154
1.51	5.112	10.83	0.000378	0.87351
1.56	5.1	10.44	0.000507	0.83914
1.61	5.109	8.601	0.000943	0.77706

Note: n=CPE-P.

Supplementary Table 5. Bader charge Calculation of nickel boride/nickel heterostructure.

——	Bader charge transfer (e^-)
Ni to Ni_3B	0.32

Bader charge calculations were performed using VASP. The result in the table is the difference between the number of electrons of Ni in pure nickel and the number of electrons of the metallic Ni component in the heterostructure. Positive value indicates the transfer of electrons from Ni to Ni_3B.

Supplementary References

1. Li, J, et al. Selective Methanol-to-Formate Electrocatalytic Conversion on Branched Nickel Carbide. *Angew. Chem. Int. Ed.* **59**, 20826-20830 (2020).
2. Li, J, et al. Superior methanol electrooxidation performance of (110)-faceted nickel polyhedral nanocrystals. *J Mater. Chem. A* **7**, 22036-22043 (2019).
3. Chinnadurai, D., Lee, S.J., Yu, Y., Nam, S.Y. & Choi, M.Y. Cation modulation in dual-phase nickel sulfide nanospheres by pulsed laser irradiation for overall water splitting and methanol oxidation reaction. *Fuel* **320**, 123915 (2022).
4. Pieta, I.S., et al. Electrocatalytic methanol oxidation over Cu, Ni and bimetallic Cu-Ni nanoparticles supported on graphitic carbon nitride. *Appl. Catal. B* **244**, 272-283 (2019).
5. Rezaee, S. & Shahrokhian, S. Facile synthesis of petal-like NiCo/NiO-CoO/nanoporous carbon composite based on mixed-metallic MOFs and their application for electrocatalytic oxidation of methanol. *Appl. Catal. B* **244**, 802-813 (2019).
6. Hou, C., Yang, W., Yang, X., Li, B., Gao, H. & Luo, X. In situ sulfidation for controllable hetero-interface engineering of α-$Ni(OH)_2$–Ni_3S_4 hybrid structures realizing robust electrocatalytic methanol oxidation. *Chem. Commun.* **56**, 5283-5286 (2020).
7. Yang, B., Yu, Y., Qiao, J., Yuan, L., Shen, X. & Hu, X. Solution plasma method for the preparation of Cu-Ni/CuO-NiO with excellent methanol electrocatalytic oxidation performance. *Appl. Surf. Sci.* **513**, 145808 (2020).
8. Dubale, A.A., et al. High-Performance Bismuth-Doped Nickel Aerogel Electrocatalyst for the Methanol Oxidation Reaction. *Angew. Chem. Int. Ed.* **59**, 13891-13899 (2020).
9. Zhang, M., et al. Synergistic Effect of Nickel Oxyhydroxide and Tungsten Carbide in Electrocatalytic Alcohol Oxidation. *Chem. Mater.* **34**, 959-969 (2022).

10. Que, R., Li, M., Yao, H., Wang, X., Liao & F., Shao, M. Unusual Effect of Trace Water on the Structure and Activity of Ni_xCo_{1-x} Electrocatalysts for the Methanol Oxidation Reaction. *ChemSusChem* **13**, 964-973 (2020).
11. Wu, N., et al. Nickel nanocrystal/nitrogen-doped carbon composites as efficient and carbon monoxide-resistant electrocatalysts for methanol oxidation reactions. *Nanoscale* **12**, 21687-21694 (2020).
12. Sheikhi, S. & Jalali, F. Remarkable electrocatalytic activity of Ni-nanoparticles on MOF-derived ZrO_2-porous carbon/reduced graphene oxide towards methanol oxidation. *Int. J. Hydrog. Energy* **46**, 10723-10738 (2021).
13. Li, M., et al. Value-Added Formate Production from Selective Methanol Oxidation as Anodic Reaction to Enhance Electrochemical Hydrogen Cogeneration. *ChemSusChem* **13**, 914-921 (2020).
14. Hao, J., et al. In situ facile fabrication of $Ni(OH)_2$ nanosheet arrays for electrocatalytic co-production of formate and hydrogen from methanol in alkaline solution. *Appl. Catal. B* **281**, 119510 (2021).
15. Guo, X., Liang, T., Zhang, D., Zhang, M., Lin, Y. & Lai, C. Facile fabrication of 3D porous nickel networks for electro-oxidation of methanol and ethanol in alkaline medium. *Mater. Chem. Phys.* **221**, 390-396 (2019).
